# A dominant negative 14-3-3 mutant in *Schizosaccharomyces pombe* distinguishes the binding proteins involved in sexual differentiation and check point

**DOI:** 10.1371/journal.pone.0291524

**Published:** 2023-10-03

**Authors:** Tomohito Ohshima, Zhang Jiajun, Takuki Fukamachi, Yuko Ohno, Hiroko Senoo, Yasuhiro Matsuo, Makoto Kawamukai

**Affiliations:** Faculty of Life and Environmental Sciences, Department of Life Sciences, Shimane University, Matsue, Shimane, Japan; Kindai University: Kinki Daigaku, JAPAN

## Abstract

The homothallic fission yeast *Schizosaccharomyces pombe* undergoes sexual differentiation when starved, but *sam* (skips the requirement of starvation for mating) mutants such as those carrying mutations in adenylate cyclase (*cyr1*) or protein kinase A (*pka1*) mate without starvation. Here, we identified *sam3*, a dominant negative allele of *rad24*, encoding one of two 14-3-3 proteins. Genetic mapping and whole-genome sequencing showed that the *sam3* mutation comprises a change in nucleotide at position 959 from guanine to adenine, which switches the amino acid at position 185 from glutamic acid to lysine (E185K). We generated the *rad24-*E185K integrated mutant and its phenotype was similar to that of the *sam3* mutant, including calcium sensitivity and UV non-sensitivity, but the phenotype is different from that of the *Δrad24* strain. While the UV-sensitive phenotype was observed in the *Δrad24* mutant, it was not observed in the *sam3* and *rad24-*E185K mutants. The expression of the *rad24*-*E185K* gene in wild type cells induced spore formation in the nutrient rich medium, confirming *rad24*-*E185K* is dominant. This dominant effect of *rad24*-*E185K* was also observed in Δ*ras1* and Δ*byr2* diploid mutants, indicating that *rad24*-*E185K* generate stronger phenotype than *rad24* null mutants. Ste11, the key transcription factor for sexual differentiation was expressed in *sam3* mutants without starvation and it predominantly localized to the nucleus. The Rad24-E185K mutant protein retained its interaction with Check point kinase1 (Chk1), whereas it reduced interaction with Ste11, an RNA binding protein Mei2, and a MAPKKK Byr2, freeing these proteins from negative regulation by Rad24, that account for the *sam* phenotype and UV non-sensitive phenotype. Glucose depletion in *rad24*-*E185K* or Δ*pka1* Δ*rad24* double mutation induced haploid meiosis, leading to the formation of spores in haploid. The position of glutamic acid 185 is conserved in all major 14-3-3s; hence, our finding of a dominant negative allele of 14-3-3 is useful for understanding 14-3-3s in other organisms.

## Introduction

The fission yeast *Schizosaccharomyces pombe* proliferates continuously when it has abundant nutrients but arrests cell cycle progression in the G1 phase when lacking nitrogen or glucose. Homothallic cells (*h*^90^) or a mixture of heterothallic cells with two opposite mating types (*h*^*-*^ and *h*^*+*^) begin conjugation under such circumstances and subsequently undergo meiosis, leading to the formation of four spores. This whole process of sexual differentiation in *S*. *pombe* has been studied extensively, revealing various elements of the process [1].

Starvation of nutrients (glucose, nitrogen, or others) is the key signal that induces sexual differentiation when cells of both mating type are present. The glucose signal is mainly transferred through the glucose receptor (Git3), which is coupled with trimeric G proteins Gpa2 (α), Git5(β), and Git11 (γ) [2]. Gpa2 activates adenylyl cyclase (Cyr1) to generate cAMP from ATP [2, 3]. Cyr1 interacts with its associated protein Cap1, which plays a regulatory role of Cyr1 [4] in addition to the role in cytoskelton. Overexpression of Cyr1 results in sterility, and the proteins that overcome this sterility, encoded by *moc1−moc4*, interact with each other to form a complex [5–9]. cAMP binds to Cgs1, a regulator of PKA [10, 11], and catalytically active protein kinase Pka1 subsequently is released to inhibit the transcription factor Rst2 [12, 13]. Under glucose starvation, the whole cAMP-PKA pathway is downregulated; hence, Rst2 remains active, allowing it to bind to the upstream region of *ste11*, encoding another transcription factor. Ste11 upregulates genes involved in meiosis including *mat1-Pm*, *mat1-Mm*, *ste6*, and *mei2* [14–16]. Mei2 is an RNA-binding protein that acts as a master regulator of meiosis to control the degradation of meiosis-specific genes [14, 17]. Ste11 is a key transcription regulator controlled in multiple ways at the level of transcription, modification, and translation [8, 18, 19]. Mei2 and Ste11 are both phosphorylated by Pat1 kinase; inhibitory phosphorylations to halt initiation of meiosis [20]. Phosphorylated Mei2 and Ste11 preferably bind with Rad24. The mating signal is recognized by the receptor coupled with Gpa1, which regulates Byr2 and subsequently regulates Byr1 and then Spk1 as a MAP kinase signaling cascade [19, 21, 22]. Spk1 regulates Ste11 as well as WD repeat protein Cpc2 and RNA-binding protein Msa2 [8]. The Ras1 protein recruits the MAPKK kinase Byr2 to the membrane [23] where it is activated [24]. Byr2 is maintained in an inactive form by binding to 14-3-3 homologs Rad24 and Rad25 [25].

We previously reported that *sam* (skips the requirement of starvation for mating) mutants [26] undergo mating without requiring nutrient starvation. They include mutants of *pka1* and *rad24* [10, 27]. The *sam4* allele is a missense mutation of *rad24*. The *msa1* and *msa2* genes [28, 29], both encoding RNA-binding proteins that negatively regulate sexual development, were identified as suppressors of *sam1*. We also found that *sla1* [30, 31] encoding a homolog of mammalian La protein and *zds1* involved in Ca^2+^ tolerance suppressed the *ras1* deletion phenotype when analyzing *sam3* and *sam9* [32], but *sam3* and *sam9* remain to be elucidated.

In the present study, we identified *sam3* and *sam9* as the dominant negative alleles of *rad24*. The *sam3* allele encodes the E185K mutated Rad24 protein responsible for the dominant negative phenotype. We found that the E185K mutation in Rad24 weakened interaction with Ste11 [20], Mei2 [33], and Byr2 [25], but not Chk1 [34], which accounts for the *sam* phenotype and UV non-sensitivity in *sam3* and *sam9* [26]. We also studied the genetic interaction between *rad24* and *pka1*.

## Materials and methods

### Strains and media

The *S*. *pombe* strains used in this study are listed in Table 1. *S*. *pombe* cells were grown in YES-rich medium (0.5% yeast extract, 3% glucose, 225 mg/L adenine, histidine, leucine, uracil, and lysine hydrochloride) or minimum synthetic medium (EMM2) supplemented with 225 mg/L adenine, leucine, and/or uracil when necessary [35]. Nitrogen-free EMM2 medium (1% glucose without ammonium chloride) was used to culture *S*. *pombe* when the mating efficiency had to be measured. *Escherichia coli* strain DH5α was used for plasmid manipulation. *E*. *coli* cells were grown in LB medium (1% polypeptone, 0.5% yeast extract, 1% NaCl, pH 7.2).

**Table 1:** Strains used in this study.

Strain	Genotype	Source
SP66	*h*^*90*^ *ade6-M210 leu1-32*	Kawamukai
SP870	*h*^*90*^ *ade6-M210 leu1-32 ura4-D1*8	Kawamukai
SP416	*h*^*90*^ *ade6-M216 sam3*	[26]
SP418	*h*^*90*^ *ade6-M216 sam4*	[26]
SP430	*h*^*90*^ *ade6-M216 sam9*	[26]
TP4-5A	*h*^*-*^ *ade6-M210 leu1-32 ura4-D18*	Kawamukai
TMS1	*h*^*90*^ *ade6-M210 leu1-32 ura4-D18 rad24*::*kanMX6*	[27]
TMS2	*h*^*-*^ *ade6-M210 leu1-32 ura4-D18 rad24*::*kanMX6*	[27]
JZ633	*h*^*90*^ *ade6-M216 leu1-32 ura4-D18 pka1*::*ura4*	Yamamoto
YA199	*h*^*90*^ *ade6-M216 his7 lys1 ura4*::*hphMX6 rec12*::*kanMX6*	Yamamoto
WY1	*h*^*-*^ *ade6-M210 rec12*:: *kanMX6 sam3*	This study
SP24U1	*h*^*90*^ *ade6-M210 leu1-32 ura4-D18 rad24*::*ura4*	[25]
SP25U1	*h*^*90*^ *ade6-M210 leu1-32 ura4-D18 rad25*::*ura4*	[25]
SPRUD	*h*^*90*^*/h*^*90*^ *ade6-M210/ade6-M210 leu1-32/leu1-32 ura4-D18/ura4-D18 ras1*::*ura4/ras1*::*ura4*	[26]
SPSUD	*h*^*90*^*/h*^*90*^ *ade6-M210/ade6-M210 leu1-32/leu1-32 ura4-D18/ura4-D18 byr2*::*ura4/byr2*::*ura4*	[26]
SPBUD	*h*^*90*^*/h*^*90*^ *ade6-M210/ade6-M210 leu1-32/leu1-32 ura4-D18/ura4-D18 byr1*::*ura4/byr1*::*ura4*	[26]
TH37	*h*^*-*^ *ura4 chk1-13myc-hphMX6*	[20]
KCR51	*h*^*-*^ *leu1-32 ura4 mei2-3HA-kanMX6*	[36]
KK1	*h*^*90*^ *ade6-M210 leu1-32 rad24-5FLAG-natMX6*	This study
KK3	*h*^*90*^ *ade6-M210 leu1-32 rad25-GFP-hphMX6*	This study
YO416	*h*^*-*^ *ade6-M216 sam3*	This study
YUK6	*h*^*90*^ *ade6-M210 his7 lys1 rec12*::*kanMX6*	This study
YUK13	*h*^*-*^ *ade6-M216 leu1-32*, *ura4*::*hphMX6 rec12*::*kanMX6 sam3*	This study
YUK17	*h*^*-*^ *ade6-M210 leu1-32 ura4-D18 ste11-GFP-kanMX6*	This study
YUK18	*h*^*-*^ *ade6-M210 leu1-32 ura4-D18 ste11-GFP-kanMX6*	This study
YUK20	*h*^*90*^ *ade6-M210 leu1-32 ura4-D18 ste11-GFP-kanMX6*	This study
YUK22	*h*^*90*^ *ade6-M210 leu1-32 ura4-D18 ste11-GFP-kanMX6 sam3*	This study
FK1	*h*^*90*^ *ade6-M210 leu1-32 rad24-E185K-kanMX6*	This study
FK2	*h*^*-*^ *ade6-M210 leu1-32 ura4-D18 rad24-E185K-kanMX6*	This study
FK3	*h*^*90*^ *ade6-M210 sam3-rad24*^*+*^*-kanMX6*	This study
HRS2	*h*^*90*^ *ade6-M210 leu1 rad24(E185K)-kanMX6 ste11-GFP-hphMX6*	This study
HRS5	*h*^*90*^ *ade6-M210 leu1-32 rad24-5FLAG-natMX6 rad25-GFP-hphMX6*	This study
HRS6	*h*^*90*^ *ade6-M210 leu1 rad24-5FLAG-natMX6 ste11-GFP-kanMX6*	This study
HRS7	*h*^*-*^ *ade6-M210 leu1-32 rad24-5FLAG-natMX6 ste11-GFP-kanMX6*	This study
HRS8	*h*^*90*^ *ade6-M210 leu1-32 rad24-5FLAG-natMX6 mei2-3HA-kanMX6*	This study
HRS12	*h*^*90*^ *ade6-M210 leu1-32 rad24-5FLAG-natMX6 chk1-13myc-hphMX6*	This study
HRS16	*h*^*90*^ *ade6-M210 rad24(E185K)-5FLAG-natMX6 chk1-13myc-hphMX6*	This study
HRS17	*h*^*90*^ *leu1-32 rad24(E185K)-5FLAG-natMX6 chk1-13myc-hphMX6*	This study
HRS26	* h*^*90*^ *ade6-M210 leu1-32 rad24(E185K)-5FLAG-natMX6 rad25-GFP-hphMX6*	This study
HRS27	*h*^*90*^ *ade6-M210 leu1-32 rad24(E185K)-5FLAG-natMX6 ste11-GFP-kanMX6*	This study
HRS30	*h*^*90*^ *ade6-M210 rad24(E185K)-5FLAG-natMX6 mei2-3HA-kanMX6*	This study
YMP27	*h*^*-*^ *leu1-32 ura4-D18 pka1-GFP(S65T)-hphMX6*	This study
YMP178	*h*^*-*^ *leu1-32 ura4-D18 pka1*::*natMX6*	This study
TOP1	*h*^*90*^ *ade6-M210 leu1-32 ura4-D18 rad24-5FLAG-natMX6*	This study
TOP2	*h*^*-*^ *ade6-M210 leu1-32 ura4-D18 rad24-5FLAG-natMX6*	This study
TOP3	*h* ^*90*^ *ade6-M210 leu1-32 ura4-D18 rad24(E185K)-5FLAG -natMX6*	This study
TOP4	*h*^*-*^ *ade6-M210 leu1-32 ura4-D18 rad24(E185K)-5FLAG -natMX6*	This study
TOP5	*h*^*90*^ *ade6-M210 leu1-32 ura4-D18 rad24*::*kanMX6 pka1*::*natMX6*	This study
TOP6	*h*^*90*^ *ade6-M210 leu1-32 ura4-D18 rad24*::*kanMX6 pka1-GFP(S65T)-hphMX6*	This study
TOP9	*h*^*90*^ *ade6-M210 leu1-32 ura4-D18 rad24(E185K)-5FLAG -natMX6 pka1-GFP(S65T)-hphMX6*	This study
TOP10	*h*^*+*^ *ade6-M210 leu1-32 ura4-D18 rad24*::*kanMX6 pka1-GFP(S65T)-hphMX6*	This study
TOP11	*h*^*+*^ *ade6-M210 leu1-32 ura4-D18 rad24(E185K)- 5FLAG-natMX6 pka1-GFP(S65T)-kanMX6*	This study
TOP12	*h*^*90*^ *ade6-M210 leu1-32 ura4-D18 byr1*::*ura4 rad24(E185K)-kanMX6/ h*^*90*^ *ade6-M216 leu1-32 ura4-D18 byr1*::*ura4*	This study
TOP13	*h*^*+*^ *ade6-M210 leu1-32 ura4-D18 rad24*::*kanMX6*	This study
TOP14	*h*^*90*^ *ade6-M210 leu1-32 ura4-D18 rad24*::*kanMX6 pka1*::*natMX6*	This study
TOP15	*h*^*90*^ *ade6-M210 leu1-32 ura4-D18 byr2*::*ura4 rad24(E185K)-kanMX6/ h*^*90*^ *ade6-M216 leu1-32 ura4-D18 byr2*::*ura4*	This study
TOP16	*h*^*90*^ *ade6-M210 leu1-32 ura4-D18 ras1*::*ura4 rad24(E185K)-kanMX6/ h*^*90*^ *ade6-M216 leu1-32 ura4-D18 ras1*::*ura4*	This study

### Genome sequencing

Genomic DNA prepared from strain SP416 (*sam3*) and SP430 (*sam9*) was sent to Hokkaido System Science Co. Ltd. for whole-genome sequencing using a next-generation sequencer. Raw sequence data (~1 Gb) were aligned with the *S*. *pombe* L972 genomic reference sequence in the Sequence Alignment/Map (SAM) format using SAMtools (http://www.htslib.org/). The file was converted to the Bam file format to visualize mutation sites using the graphic viewer Tablet (https://ics.hutton.ac.uk/tablet/).

### Plasmids

Plasmids pREP42-rad24, pREP42-rad25, and pSLF272U-Byr2 were previously described [25]. pREP42-rad24-E185K was constructed by amplifying rad24-E185K mutant DNA with primers rad24-PrimerF (*Sal*Ⅰ) and rad24-PrimerR (*Bam*HⅠ), and subsequent cloning of the PCR fragment into pREP42. Plasmids pREP41-rad24 and pREP41-rad24-E185K were similarly constructed.

### Strain construction

The *rad24*-E185K integrated strain was constructed by amplifying the *rad24*-E185K mutated gene using primers rad24-A, rad24-W, rad24-E185K-F, and rad24-E185K–R, and the kanMX6 fragment was separately amplified from pFA6a-kanMX6 [37] using primers rad24-B1 and rad24-Z. These two PCR fragments were jointly amplified using rad24-A and rad24-Z and the product was introduced into the SP66 and TP4-5A strains to obtain stable G418 resistant transformants. Correct replacement was checked by colony-directed PCR using primers Rad24-check and nb2, and the presence of the mutation in *rad24* was verified by amplifying the *rad24* region and sequencing. The resulting strains were named FK1 (*h*^90^ *rad24*-E185K-kanMX6) and FK2 (*h*^-^ *rad24*-E185K-kanMX6). Similarly, rad24-FLAG tagging and rad24-E185K FLAG tagging strains were constructed by integrating the PCR product amplified from chromosomal DNA and pFA6a-5FLAG-natMX6 using primers rad24-A, rad24-W, rad24-B2, and rad24-Z [38]. To express Ste11-GFP, the PCR product was amplified from chromosomal DNA and pFA6a-GFP(S65T)-kanMX6 using primers ste11-w, ste11-X, ste11-Y, and ste11-Z, and the resulting PCR product was chromosomally integrated into wild type (WT) TP4-5A and YO416 (*h*^-^ *sam3*) strains, and the resulting strains were named YUK17 (*h*^-^ *ste11-GFP*) and YUK18 (*h*^-^ *sam3 ste11-GFP*). YUK20 (*h*^90^ *ste11-GFP*) and YUK21 (*h*^90^ *sam3 ste11-GFP*) strains were derived by crossing these strains with SP870A. These *ste11-GFP* expressing strains were used to derive HRS7 (*ste11-GFP* rad24-FLAG) and HRS27 (*ste11-GFP* rad24-E185K-FLAG) strains by crossing with appropriate strains. The TH37 strain expressing Chk1-Myc [36] and the KCR51strain expressing Mei2-HA [20] were used to construct *rad24*-FLAG and rad24-E185K-FLAG tagging strains by crossing with appropriate strains. These were selected for antibiotic resistance and nutrient requirements. To generate the YUK13 (*sam3 rec12*::*kanMX6*) strain we obtained the YA199 (*rec12*:: *kanMX6*) strain from M. Yamamoto and crossed it with the YOK416 (*sam3*) strain, and selected transformants on appropriate media.

### Mating and sporulation efficiency assay

Mating and sporulation efficiency was calculated using the following equation:

Mat(%)=(2Z+2A+0.5S)/(H+2Z+2A+0.5S)

where *Z* stands for the number of zygotes, *A* for the number of asci, *S* for the number of free spores, and *H* for the number of cells that failed to mate.

### Western blotting

Western blotting was conducted as described previously [39]. Briefly, ~ 1×10^8^ *S*. *pombe* cells were harvested after growth in appropriate medium, washed twice with dH_2_O, dissolved in 100 μl of dH_2_O, and samples were boiled at 95°C for 5 min. Subsequently, 120 μL of 2× Laemmli buffer (1M Tri-HCl pH6.8 (12%v/v), 2-mercaptoethanol (12% v/v), 10% SDS (40% v/v), glycerol (20% v/v), Bromohenol blue (0.02%w/v) and urea (48% w/v)) [40] was added and samples were vigorously vortexed with zirconia/silica beads for 3 min. The zirconia/silica beads and large debris were removed by centrifugation at 16,000 × g for 10 min. Each sample was subjected to SDS-PAGE and proteins were transferred to an Immobilon transfer membrane (Millipore, City, State, Country). The membrane was incubated with antibodies diluted 1:2000 in 5% skim milk in PBS-T (137 mM NaCl, 8 mM Na_2_HPO_4_·12H_2_O, 2.7 mM KCl, 1.5 mM KH_2_PO_4_, 0.1% Tween-20). The membrane was washed with PBS-T for 15 min and 5 min twice per wash, then incubated with horseradish peroxidase-conjugated anti-rabbit secondary antibody (Bio-Rad Laboratories Inc.) diluted 1:3000 in 5% skim milk in PBS-T. Secondary antibodies were detected using a ECL system as described by the manufacturer (PerkinElmer). Mouse monoclonal anti-Myc (diluted 1:1000) and rabbit polyclonal anti-PSTAIRE (Cdc2; diluted 1:1000) antibodies were purchased from Santa Cruz Biotechnology (City, State, Country). Horseradish peroxidase-conjugated anti-mouse IgG (Santa Cruz Biotechnology) or anti-rabbit IgG antibody (Promega) were used as secondary antibodies. ImageJ software (https://imagej.nih.gov/ij/download.html) was used for quantification of protein bands.

### Immunoprecipitation

Cells were grown in EMM or YES to an optical density at 600 nm of ~0.5, harvested by centrifugation, and washed with stop buffer (150 mM NaCl, 50 mM NaF, 10 mM EDTA, 1 mM NaN_3_, pH 8.0). Cells were resuspended in lysis buffer (50 mM Tris pH 8.0, 150 mM NaCl, 1% Nonidet-P40, 5 mM EDTA, 10% glycerol, 1 mM PMSF, 5 μg/mL pepstatin, 5 μg/mL leupeptin, 5 μg/mL aprotinin) and ground by vortexing with zirconia/silica beads for 4 × 15 s. The supernatant of the cell extract was prepared by centrifugation (2500 rpm for 10 min at 4°C). Cell extract (1 μg in lysis buffer) was incubated with 40 μL of Anti-FLAG(M2) beads (Sigma-Aldrich) overnight at 4°C. Beads were washed five times with 0.5 mL of phosphate-buffered saline (PBS; 137 mM NaCl, 8.1 mM Na_2_HPO_4_, 2.68 mM KCl, 1.47 mM KH_2_PO_4_), and samples were resuspended in loading buffer. After boiling for 5 min, samples were separated by SDS-PAGE using 10% polyacrylamide gels and transferred to an Immobilon transfer membrane (Millipore). Immunoblotting was performed with anti-GFP monoclonal antibody (Roche), anti-HA monoclonal antibody (Santa Cruz Biotechnology), and anti-Myc monoclonal antibody (Santa Cruz Biotechnology) as primary antibodies, and with HRP-conjugated goat anti-rabbit IgG or anti-mouse IgG (Santa Cruz Biotechnology) as secondary antibodies, together with ECL reagent (PerkinElmer).

### Microscopy observation

*S*. *pombe* cells were grown in YES liquid medium or in an appropriate medium typically to the mid-log phase at 30°C. Ste11-GFP-tagged strains were visualized in living cells, and images were captured by a BX51 microscope (Olympus) equipped with a DP74 digital camera (Olympus).

### Reproducibility and statistics

Experiments were performed at least in duplicate, and average values and standard deviation (SD) were calculated. Data from controls and tested samples were compared using two-sample *t*-tests and *p* <0.05 was considered statistically significant.

## Results

### Mapping the *sam3* locus and whole-genome sequencing of the *sam3* mutant

**Fig 1:**
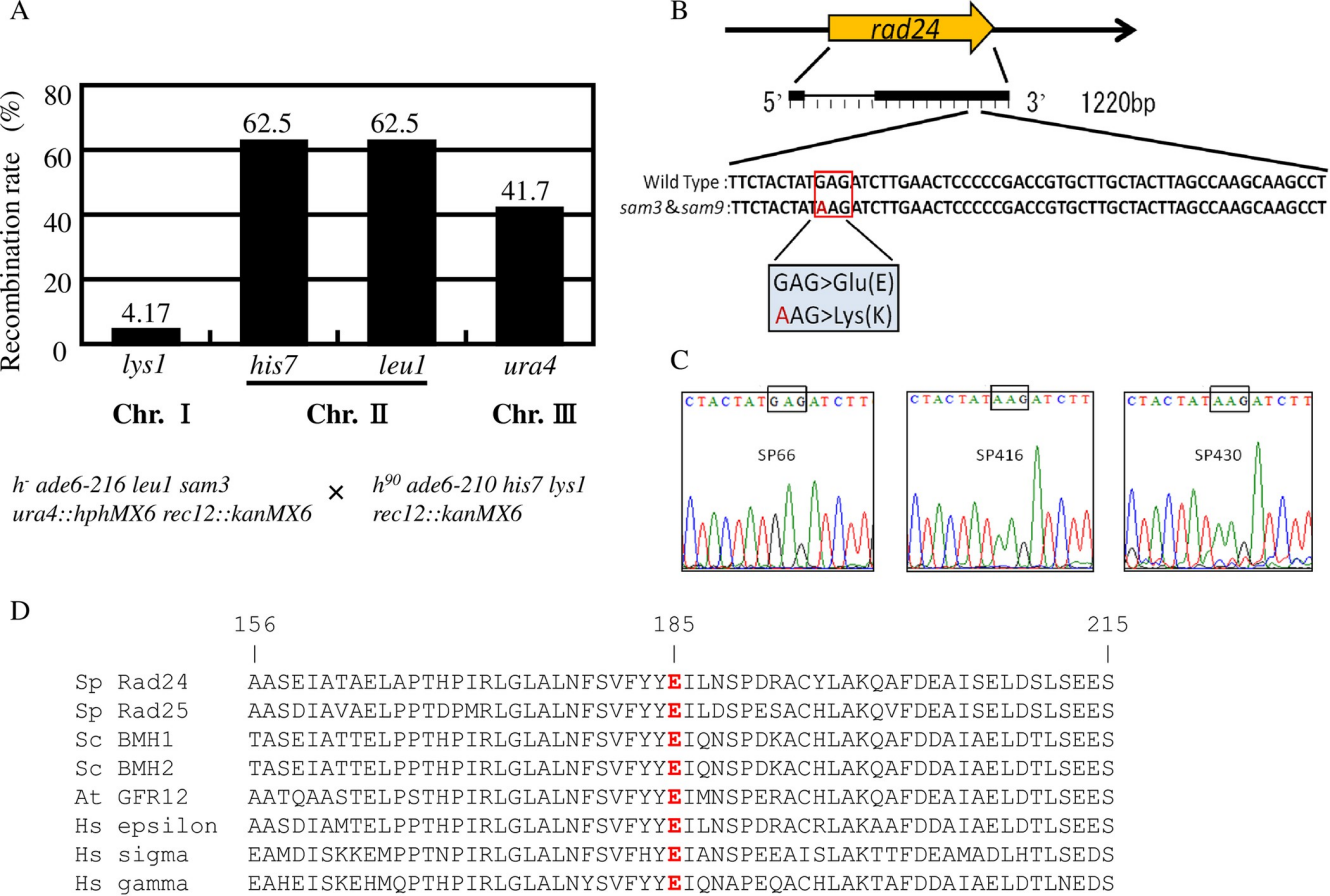
Mapping and sequencing of the mutation sites of *sam3* and *sam9*. (A) Chromosomal mapping of the mutation site of *sam3*. Mapping was conducted by crossing YUK13 (*h*^*-*^ *ade6-216 leu1 ura4*::*hphMX6 rec12*::*kanMX6 sam3*) and YUK6 (*h*^*90*^ *ade6-210 his7 lys1 rec12*::*kanMX6*) strains, yielding random spores. Spores were first grown on YES, then on YES containing CaCl_2_. The CaCl_2_-sensitive phenotype of *sam3* was co-segregated with lysine auxotrophy, caused by *lys1* mutation on Chr I, at a very low ratio (4.17%), while it was co-segregated normally with histidine auxotrophy (caused by *his7* mutation on Chr II) and leucine auxotrophy (caused by *leu1* mutation on Chr II), and uracil auxotrophy (caused by *ura4* mutation on Chr III). (B) Whole-genome sequencing of SP416 (*sam3*) and SP430 (*sam9*) mutants reveals a common mutation in *rad24* in *sam3* and *sam9* mutants. Nucleotide G959 of the *rad24* gene was changed to A, which changed glutamic acid residue 185 of Rad24 to lysine. (C) Sanger sequencing verification of the mutation sites of the *rad24* genes of SP66 (WT), SP416 (*sam3*), and SP430 (s*am9*) strains. (D) Alignment of various 14-3-3s orthologues. Glu185 is conserved in all typical 14-3-3s including *S*. *pombe* Rad25, *S*. *cerevisiae* Bmh1 and Bmh2, *Arabidopsis* GFR12, and *Homo sapiens* 14-3-3 epsilon, sigma, and gamma.

In our previous work we analyzed *S*. *pombe sam* mutants that underwent mating and subsequent sporulation on nutrient-rich medium under conditions in which WT cells scarcely mate [10, 26, 27]. We showed that *sam3* and *sam9* were the dominant mutants while others were recessive [26, 27]. The six *sam* mutants (*sam1−3* and *sam5−8*) are mutant alleles of *pka1* [10, 11] and *sam4* is a *rad24* nonsense mutant [27], but we did not succeed in unveiling the *sam3* and *sam9* mutations (S1 Fig in S1 File.). To identify the mutation site of *sam3*, we first conducted chromosomal mapping of the *sam3* mutation locus using the *rec12* deletion mutant in which meiotic chromosomal recombination is abolished, resulting in mutation of homologous chromosomes strongly maintained in parental chromosomes after meiosis. We generated the *rec12* deletion strain in the *sam3* mutant and the resulting YUK13 strain (*h*^*-*^ *ade6-216 leu1 sam3 ura4*::*hphMX6 rec12*::*kanMX6*) was crossed with the YUK6 strain (*h*^*90*^ *ade6-210 his7 lys1 rec12*:: *kanMX6*). From this diploid, spores were randomly separated and CaCl_2_-sensitive cells, which displayed the *sam3* phenotype, were selected and tested for histidine, lysine, leucine, and uracil auxotrophy. Among the 48 lines analyzed, 30 were histidine and leucine auxotrophic, 20 were uracil auxotrophic, and 2 were lysine auxotrophic (Fig 1A). Given the mutations of *lys1*, *his7*, *leu1*, and *ura4* on chromosomes I, II, II, and III, respectively, we concluded that the *sam3* mutation is located on chromosome I because *sam3* was co-segregated with the *lys1* mutation only at a very low frequency.

We then sequenced the whole genome of the *sam3* mutant and although ~20 mutations were found in the whole chromosome, chromosome I only contained six mutations. Among them, we speculated that the most relevant mutation was the change at position 3385227 from G to A, located in the *rad24* gene. This changes nucleotide 959 of *rad24*, switching glutamic acid to lysine at amino acid 185 of the Rad24 protein (Fig 1B and 1C). Whole-genome sequencing of the *sam9* mutant was also conducted at the same time, and we found the same 3385227 G to A mutation in *rad24*, although we also found different mutations in other genes. In our previous analysis we detected Rad24 proteins in *sam3* and *sam9* mutant strains, hence this mutation did not abolish expression of Rad24 [27]. We previously found that the phenotypes of *sam3* and *sam9*, including sensitivity to CaCl_2_ and KCl, were indistinguishable [26]. Therefore, we concluded that the E185K mutation of *rad24* in *sam3* and *sam9* is common and responsible for their phenotypes. The position of E185 in Rad24 is highly conserved in typical 14-3-3 proteins including *S*. *pombe* Rad25, *Saccharomyces cerevisiae* Bmh1 and Bmh2, *Arabidopsis thaliana* GFR12, and *Homo sapiens* 14-3-3s (Fig 1D).

### Phenotypes of the *rad24-E185K* mutant and *sam3*

**Fig 2:**
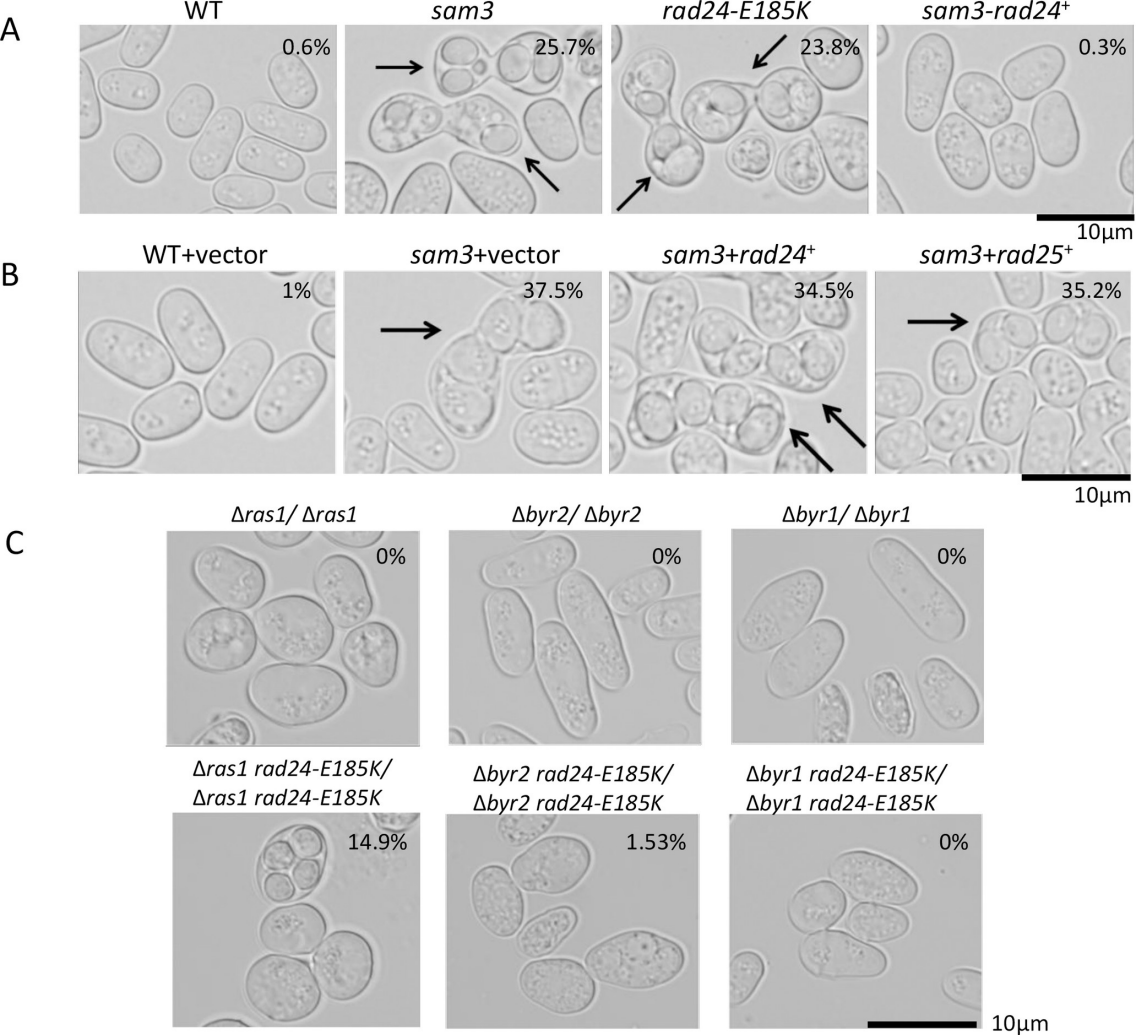
Dominant negative effect of *sam3*. (A) Mating efficiency in SP66 (WT), SP416 (*sam3*), FK1 (*rad24*-E185K), and FK3 (*sam3*- *rad24*^*+*^) strains. Mating efficiency was measured by counting 300 cells growing on YES-rich medium for 3 days. FK3 was derived by replacing the *sam3* mutant with WT *rad24*. (B) Cell morphology and mating efficiency of WT and *sam3* strains harboring the vector and a plasmid expressing *rad24* or *rad25*. Mating efficiency of WT and *sam3* stains harboring plasmids was measured by counting 300 cells growing on EMML medium for 4 days. No clear reversion of the mating and sporulation efficiency phenotype was observed following expression of *rad24* or r*ad25*. (C) Effects of deleting *ras1*, *byr2*, and *byr1* on the *rad24-E185K* mutant. Cells of SPRUD (Δ*ras1/*Δ*ras1*), SPSUD (Δ*byr*2*/*Δ*byr*2), SPBUD (Δ*byr1/*Δ*byr1*), TOP16 (Δ*ras1 rad24-E185K/*Δ*ras1 rad24-E185K*), TOP15(Δ*byr*2 *rad24-E185K /*Δ*byr*2 *rad24-E185K*), and TOP12 (Δ*byr1 rad24-E185K /*Δ*byr1 rad24-E185K*) strains were grown on EMMAL plates for 2 days. Sporulation rates of diploid strains were measured by counting 300 cells. Averages (%) of three experiments are shown.

To further confirm that the *rad24-E185K* mutation in *sam3* is solely responsible for its phenotype, we introduced the *rad24*-E185K mutation on the WT chromosome, yielding FK1 (*h*^*90*^ *rad24*-185K) and FK2 (*h*^*-*^ *rad24*-185K) strains. We then observed the phenotypes of *rad24*-185K mutants and compared them with WT, *sam3*, and Δ*rad24* strains. The cell length of heterothallic strains of L972 (*h*^-^), TMS2 (*h*^-^ Δ*rad24*), YO416 (*h*^-^ *sam3*), and FK2 (*h*^-^ *rad24-E185K*) were measured and they were 9.36 ± 2.23 μm, 7.69 ± 1.81 μm, 7.36 ± 1.9 μm, and 6.67 ± 1.75 μm, respectively (S2 Fig in S1 File.). The cell length was clearly shorter for Δ*rad24*, *sam3*, and *rad24-E185K* strains than for the WT strain, which is one of the characteristics of *rad24* defective mutants. The homothallic SP416 (*sam3*) and FK1 (*rad24*-185K) mutants mated and sporulated with high efficiency on rich medium (25.7 ± 1.2% and 23.8 ± 1.3%, respectively). When we replaced the *sam3* locus with WT *rad24*, the mating ratio clearly dropped to 0.3 ± 0.1%, comparable with that of the WT strain (0.6 ± 0.1%; Fig 2A). We expressed *rad24* or *rad25* on a plasmid in *sam3* mutants to investigate the effects of these genes on mating. The mating ratios of *sam3* and strains harboring a vector expressing *rad24* or *rad25* were 37.5 ± 2.5%, 34.5 ± 3.3%, and 35.2 ± 3.5% respectively, while that of the WT strain was 1.0 ± 0.3%. The fact that expression of *rad24* or *rad25* did not alter the mating ratio of the *sam3* mutant indicates that *sam3* gives a clear dominant phenotype (Fig 2B). We also expressed *rad24*-E185K in WT and *Δrad25* strains and found that it significantly increased the mating ratio (S3 Fig in S1 File.), confirming that *rad24-*E185K had a dominant negative effect in WT and *Δrad25 rad24*^*+*^ cells.

**Fig 3:**
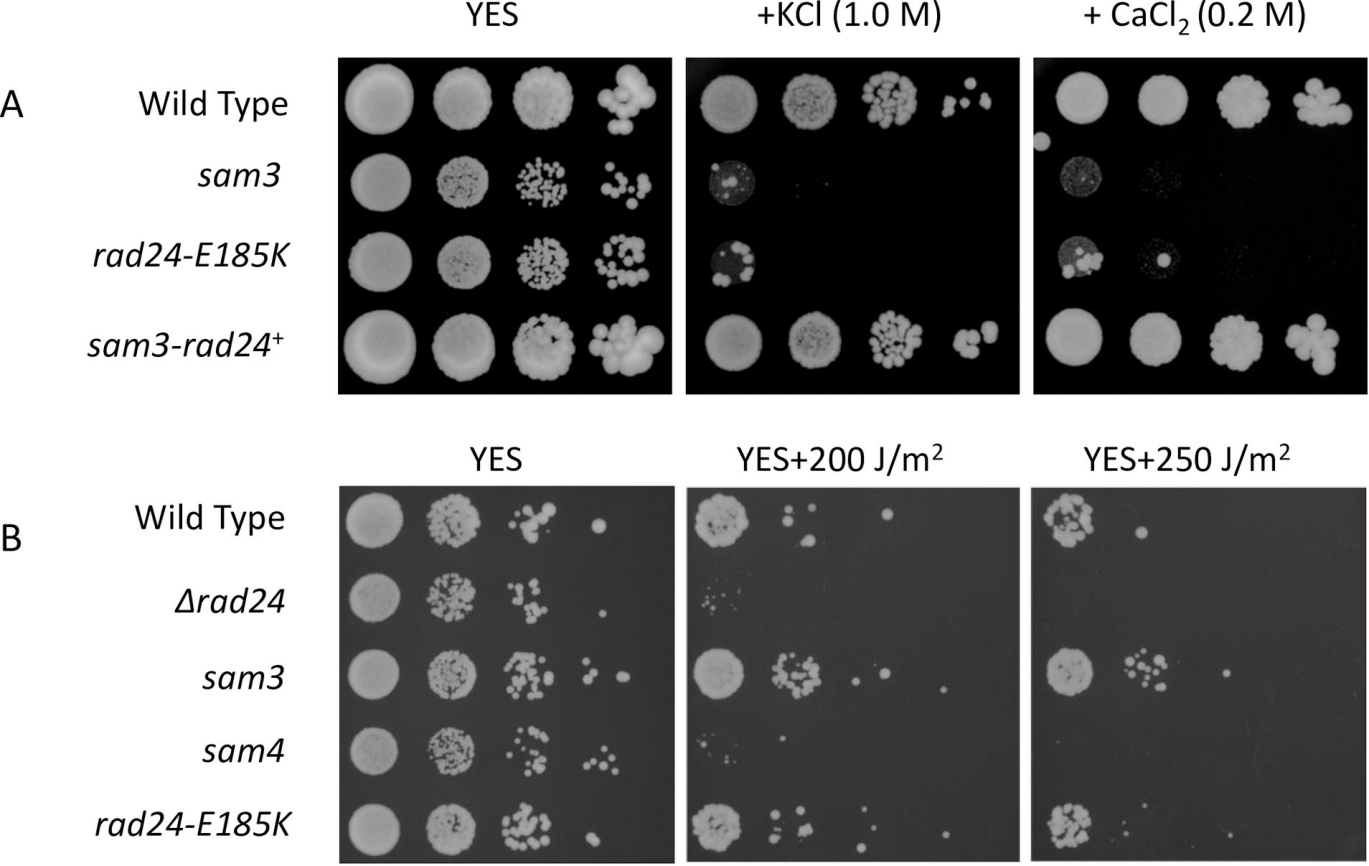
KCl, calcium, and UV sensitivity of *sam3* and *rad24*-E185K strains. (A) SP870 (WT), SP416 (*sam3*), FK1 (*rad24*-*E185K*), and FK3 (*sam3-rad24*^+^) cells were cultured at 30°C in liquid medium until log phase, concentrated to 1×10^7^ cells/mL, and then used to generate a 10-fold dilution series. Diluted cells were spotted onto YES plates and incubated at 30°C for 3 days in the presence of 1 M KCl or 0.2 M CaCl_2_. (B) SP870 (WT), TMS1 (*Δrad24*), SP416 (*sam3*), SP418 (*sam4*), and FK1 (*rad24*-*E185K*) strains were grown and used to generate a 10-fold dilution series. Cells were exposed to 0, 200, or 250 J/m^2^ UV, spotted onto YES plates, and incubated at 30°C for 3 days.

We created *ras1*, *byr1*, and *byr2* deletion strains also carried the *rad24-*E185K mutation. In these haploid strains there was no mating at all; hence, we generated diploid cells. The *rad24-*E185K mutation allowed the formation of spores in diploid *ras1* and *byr2* deletion mutants at a ratio of 14.9 ± 2.1% and 1.53 ± 0.6%, respectively, but not in *byr1* deletion diploid mutants (0%; Fig 2C). This phenotype is consistent with our previous observation in *sam3* mutants with combined deletions of *ras1*, *byr1*, and *byr2* [26]. The *sam3* and *rad24-*E185K mutants were sensitive to growth on KCl- and calcium-containing medium, but were not sensitive to UV, while *sam4* (nonsense mutant of *rad24*) and *Δrad24* strains were UV sensitive (Fig 3; see also Fig 7). Both *Δrad24* and *rad24-*E185K mutants grew slower than WT on YES medium. The phenotypes of *sam3* and *rad24-*E185K mutants were indistinguishable. Based on these results we concluded that *sam3* is the allele of *rad24-*E185K. We also found that while the *Δrad24* strain was sensitive to growth on 5 μM HU-containing medium, the *rad24-*E185K mutant grew well under these conditions, like the WT strain (S4 Fig in S1 File.). This phenomenon is similar to the UV insensitive phenotype of *rad24-*E185K. The *rad24-*E185K mutant and the *Δrad24* strain were both sensitive to growth on 15 μg/mL microtubule-destabilizing drug thiabendazole (TBZ)-containing medium under conditions in which WT and *Δrad25* strains grew well.

### Expression of Ste11 in the *rad24*-E185K mutant and *sam3*

**Fig 4:**
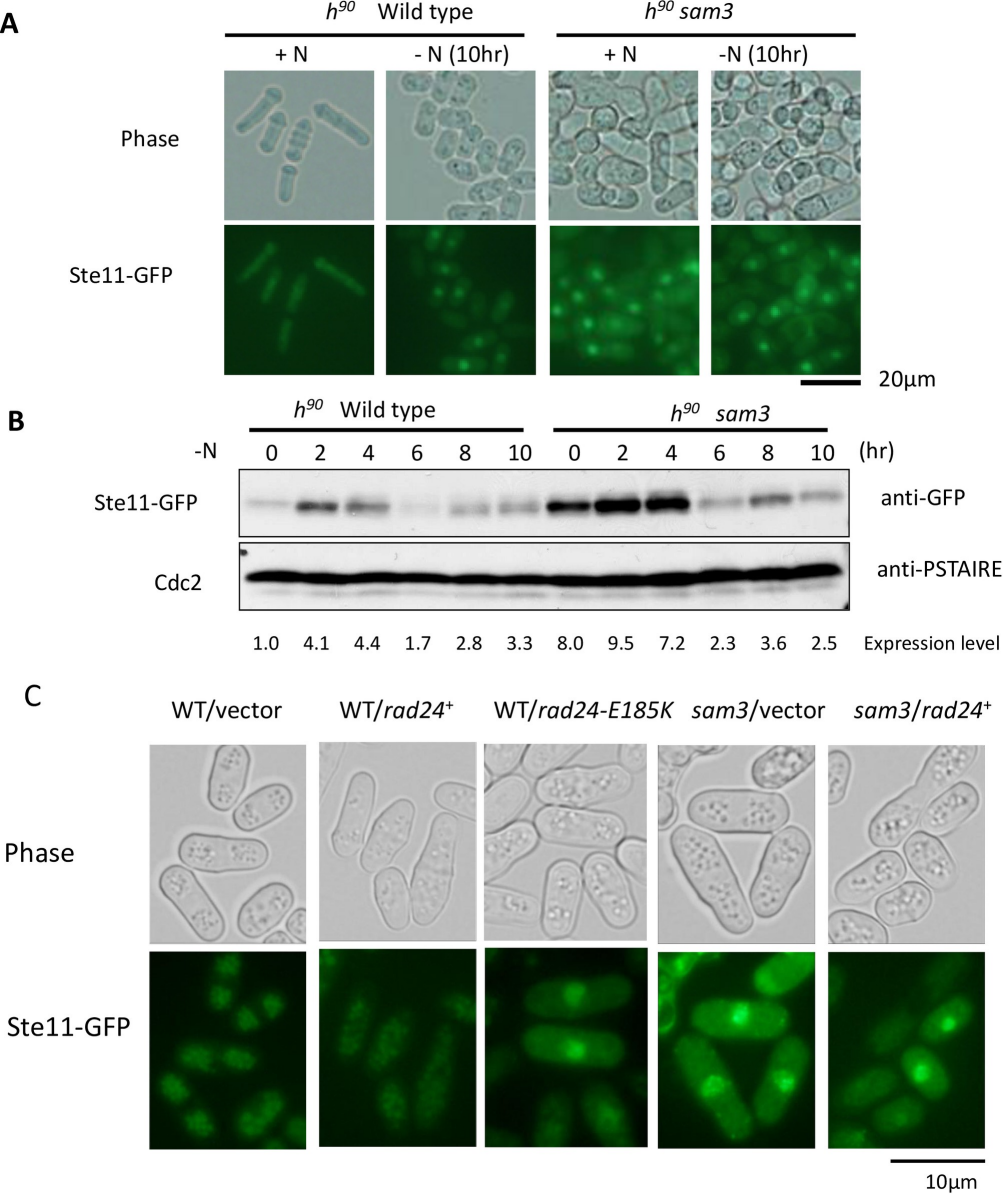
Expression and localization of Ste11 in the *sam3* mutant. (A) Localization of Ste11 in homothallic YUK20 (WT *ste11*-*GFP*) and YUK22 (*sam3 ste11*-*GFP*) strains before and after nitrogen starvation for 10hr. (B) YUK20 (WT) and YUK22 (*sam3*) strains were grown under nitrogen-starved conditions for 10 h and Ste11 expression was monitored by western blotting using anti-GFP antibody. The relative intensity of Ste11 bands was adjusted against the Cdc2 signal quantified by ImageJ (shown at the bottom). (C) Localization of Ste11 was monitored in the YUK20 (WT) strain harboring vector or a plasmid pREP42-rad24 expressing *rad24* and pREP42-rad24-E185K expressing *rad24-E185K*, and in the YUK22 (*sam3*) mutant harboring vector or pREP42-rad24. Cells were grown in EMMAL medium for 24 h and Ste11 levels in the nucleus were determined upon expression of *rad24-E185K*.

We next investigated the expression level and localization of Ste11 in the *sam3* mutant. Ste11 was localized to the cytoplasm in WT cells before nitrogen starvation and in the nucleus after nitrogen starvation, but Ste11 was localized to the nucleus in the *sam3* mutant even before nitrogen starvation (Fig 4A). In the WT strain Ste11 was expressed inductively upon nitrogen starvation after 2 h, but it was constitutively expressed without starvation in the *sam3* mutant (Fig 4B). Expression of *rad24* did not alter the nuclear localization of Ste11 in *sam3*, while Ste11 was localized to the cytoplasm in WT cells (Fig 4C). Expressing the *rad24-E185K* mutant on a plasmid resulted in Ste11 localization to the nucleus; hence, ectopic expression of the *rad24*-E185K mutant restricted the nuclear localization of Ste11 (Fig 4C). Furthermore, the *rad24-E185K* mutation conferred a clear dominant negative phenotype on WT cells.

### Interaction of Rad24-E185K with Chk1

**Fig 5:**
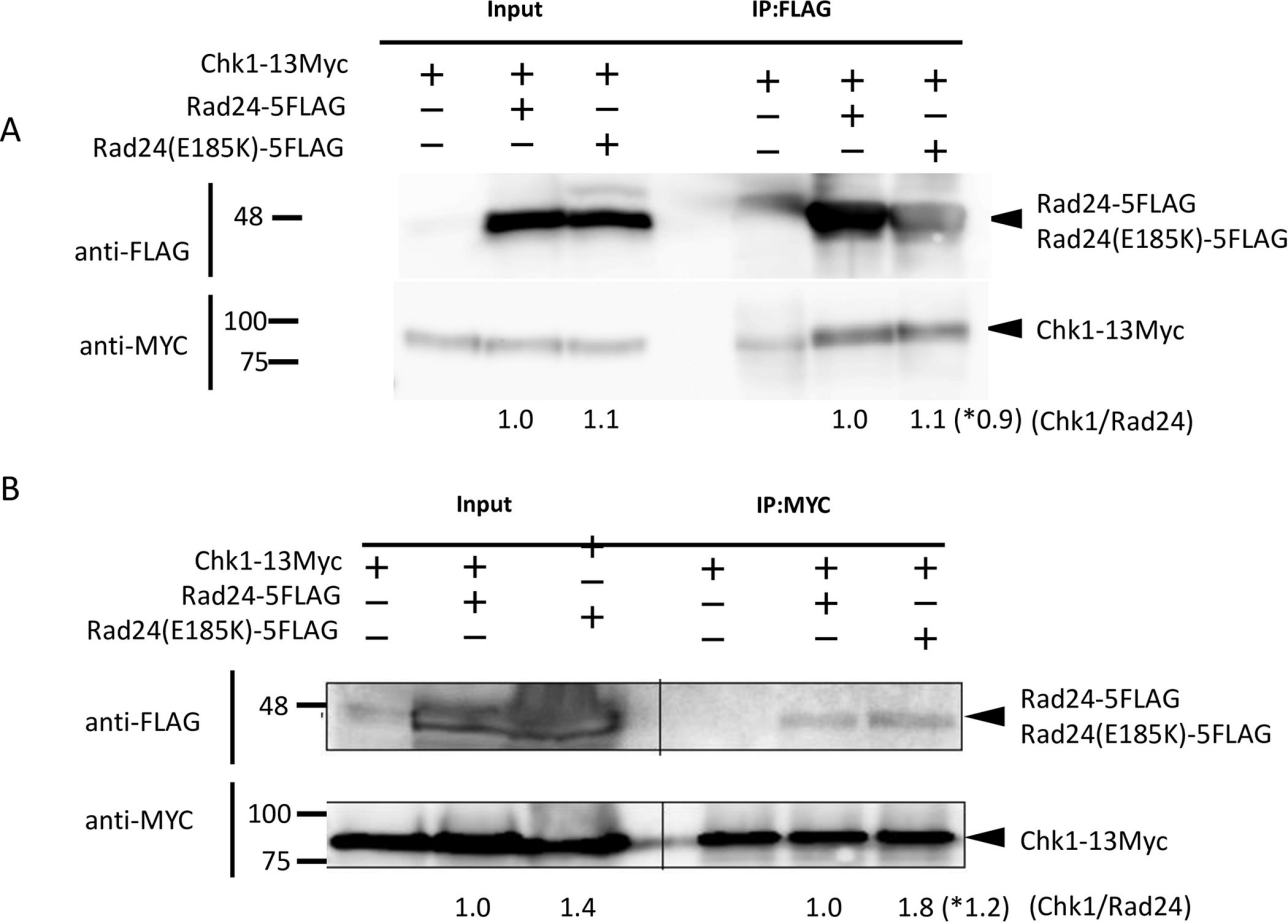
Immunoprecipitation of Chk1 with Rad24 and Rad24-E185K. (A) Cells of HRS12 (Rad24-5FLAG Chk1-13Myc) and HRS17 (Rad24-E185K-5FLAG Chk1-13Myc) were grown on YES medium to log phase, harvested, and disrupted. Rad24-5FLAG or Rad24 (E185K) protein was pulled down by FLAG-conjugated beads, and Chk1-13Myc was detected using anti-Myc antibody. The left panel indicates the input. (B) Reciprocally, using HRS12 and HRS17 strains, Chk1-13Myc was immunoprecipitated with anti-Myc antibody and detected using anti-FLAG antibody. The strengths of the Chk1, Rad24, and Rad24E185K bands were quantified by ImageJ, and the relative intensity of Chk1 vs. Rad24E185K compared with Chk1 vs. Rad24 was calculated (shown at the bottom). *Numbers indicate the relative fold change of Input / IP samples.

As indicated above, the Rad24-E185K mutant showed CaCl_2_-sensitive but not UV-sensitive phenotypes, while *rad24* deletion strains showed sensitivity to both CaCl_2_ and UV. We therefore explored the reasons for the difference between Δ*rad24* and *rad24*-E185K strains with respect to UV sensitivity. We hypothesized that this difference may be caused by a lack of Chk1 activation in the *rad24* deletion strain unlike the Rad24-E185K mutant because Chk1 is known to interact with Rad24 [34, 41]. We therefore tested the interaction of the Rad24-E185K mutant with Chk1. Rad24 and Rad24-E185K were fused with FLAG, and Chk1 was fused with Myc. Cell extracts from the HRS16 strain expressing Rad24-FLAG and Chk1-13Myc were immunoprecipitated with FLAG antibody-conjugated beads and western blotted with anti-Myc antibody. Rad24 was detected with FLAG antibody and Chk1 was detected with anti-Myc antibody. Rad24-FLAG was clearly immunoprecipitated with Chk1-Myc, and Rad24-E185K was also immunoprecipitated with Chk1-Myc, but the difference with respect to Rad24-FLAG (0.9-fold) was slight (Fig 5). Reciprocally, Chk1-13Myc was immunoprecipitated with Myc antibody and detected with FLG antibody. The interaction of Chk1 with Rad24-E185K was only slightly higher (1.2-fold) than that with Rad24. Similarly, interaction of Rad25 with Rad24 and Rad24-E185K was tested by immunoprecipitation (S5 Fig in S1 File.). There was no clear difference (1.2-fold) between the interactions of Rad24 and Rad24-E185K with Rad25, suggesting the *sam3* mutant contains a heteromeric form of Rad25 and Rad24-E185K in addition to the Rad25 homomer.

### Interaction of Rad24-E185K with Ste11, Mei2, and Byr2

**Fig 6:**
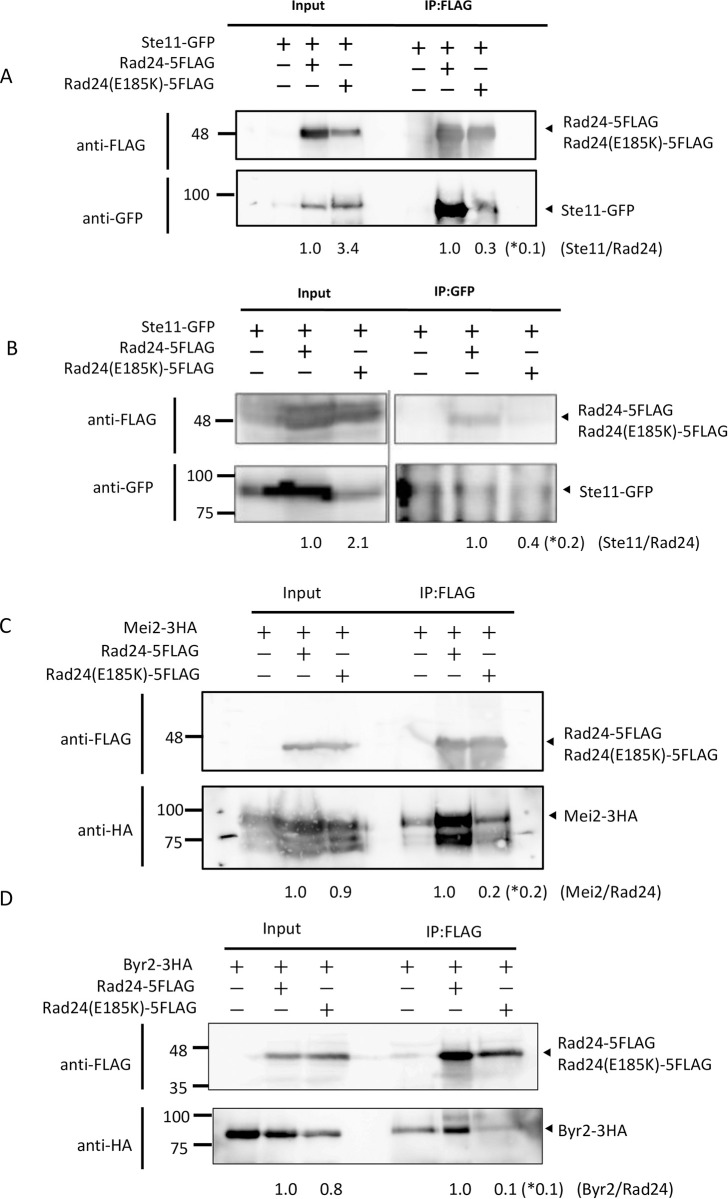
Immunoprecipitation of Ste11, Mei2, and Byr2 with Rad24 and Rad24-E185K. (A) HRS7 (Rad24-5FLAG Ste11-GFP) and HRS27 (Rad24-FLAG Ste11-GFP) cells were grown on YES medium to log phase. Rad24-5FLAG and Rad24-E185K-5FLAG protein was pulled down by FLAG-conjugated beads, and Ste11-GFP was detected using anti-GFP antibody. The strengths of the Ste11, Rad24, and Rad24E185K bands were quantified by Image J, and the relative intensity of Ste11 vs. Rad24E185K against Chk1 vs. Rad24 was calculated (shown at the bottom). *Number indicates Input / IP samples. (B) Reciprocally, using HRS7 and HRS27 strains, Ste11-GFP immunoprecipitated with anti-GFP antibody and Rad24-5FLAG or Rad24-E185K-5FLAG protein was detected using anti-FLAG antibody. Numbers were calculated as in (A). (C) Cells of HRS8 (Rad24-5FLAG Mei2-3HA) and HRS30 (Rad24-E185K-5FLAG Mei2-3HA) were grown on YES medium to log phase. Rad24-5FLAG or Rad24-E185K-5FLAG protein was pulled down by FLAG-conjugated beads and Mei2 was detected using anti-HA antibody. Numbers were calculated as in (A). (D) Cells of the TOP2 (Rad24-5FLAG) strain harboring plasmid pSLF272U-B2 and the TOP4 (Rad24-E185K-5FLAG) strain harboring plasmid pSLF272U-B2 were grown on EMMAL. Rad24-5FLAG or Rad24-E185K-5FLAG protein was pulled down by FLAG-conjugated beads and Byr2 was detected using anti-HA antibody. Numbers were calculated as in (A).　*Numbers indicate the relative fold change of Input / IP samples after subtracting the background band intensity.

Regarding sexua1 development, at least three proteins, Ste11, Mei2, and Byr2, have been shown to interact with Rad24 [20, 25]. We therefore tested the interaction of the Rad24-E185K mutant with these proteins. The HRS7 strain expressing Rad24-5FLAG and Ste11-GFP, and the HRS27 strain expressing Rad24-E185K-5FLAG and Ste11-GFP were used to pull down Rad24 or Rad24-E185K protein using FLAG antibody-conjugated beads, and samples were detected by western blotting using anti-GFP antibody (Fig 6A). The immunoprecipitated protein band of Ste11-GFP was weaker (0.1-fold) in the Rad24-E185K mutant than in Rad24-expressing cells. Reciprocally, Ste11-GFP was immunoprecipitated with anti-GFP antibody and Rad24-FLAG or Rad24-E185K-FLAG was detected using anti-FLAG antibody (Fig 6B). The Ste11-GFP band was clearly weaker (0.2-fold) in the Rad24-E185K-5FLAG pulldown sample than in the Rad24-5FLAG sample. To test the interaction of Mei2 with Rad24 and Rad24-E185K, cell extracts of the HRS8 strain expressing Mei2-3HA and Rad24-5FLAG, and the HRS30 strain expressing Mei2-3HA and Rad24-E185K-5FLAG were pulled down with FLAG antibody-conjugated beads and western blotted using anti-HA antibody (Fig 6C). The amount of immunoprecipitated Mei2-3HA protein was lower (0.2-fold) in the Rad24-E185K mutant than the Rad24 strain. To test the interaction of Byr2 with Rad24 and Rad24-E185K, we expressed *byr2* on a plasmid because expression of *byr2* under the control of its own promoter is very low. The TOP2 (Rad24-5FLAG) strain harboring plasmid pSLF272U-B2 and the TOP4 (Rad24-E185K-5FLAG) strain harboring plasmid pSLF272U-B2 were used. Rad24 or Rad24-E185K proteins from those cells were pulled down with FLAG antibody-conjugated beads and tested by western blotting with anti-HA antibody. The immunoprecipitated band of Byr2-3HA was weaker (0.1-fold) in TOP4 (Rad24- E185K-FLAG) than in TOP2 (Rad24-FLAG) cells (Fig 6D). Thus, interaction of Byr2 with Rad24 was weakened by mutation of E185K. Therefore, interactions of Ste11, Mei2, and Byr2, all involved in sexual development, with Rad24-E185K were weaker than those with Rad24.

### Phenotypes of *pka1 rad24* double deletion mutants

**Fig 7:**
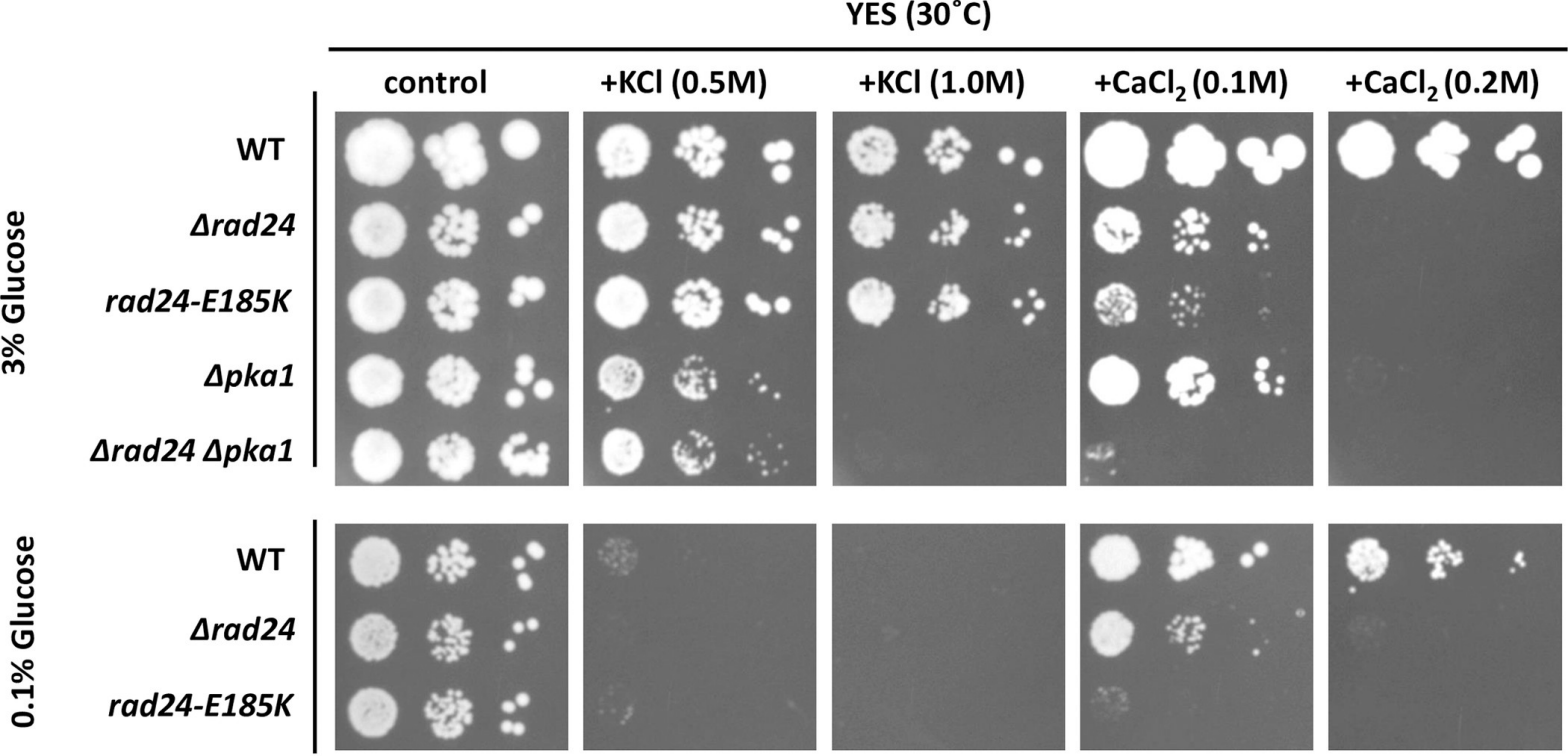
KCl and calcium sensitivity of *rad24 pka1* mutants. Cells of TP4-5A (*h*^*-*^ WT), TOP13 (*h*^*-*^ Δ*rad24*), TOP4 (*h*^*-*^ *rad24-E185K*), YMP178 (*h*^*-*^ Δ*pka1*), and TOP14 (*h*^*-*^ Δ*rad24* Δ*pka1*) were spotted onto YES plates following serial dilution and incubated at 30°C for 3 days on YES medium in the presence of 0.5 M KCl, 1 M KCl, 0.1 M CaCl_2_, or 0.2 M CaCl_2_. YES medium containing 0.1% glucose was used as a control (lower panel).

Since *pka1* and *rad24* have been identified as *sam* mutants involved in regulating sexual differentiation, we next constructed *Δpka1 Δrad24* double mutants and tested their phenotypes. *S*. *pombe Δrad24* cells were sensitive to growth on medium containing 0.1 M CaCl_2_, and this phenotype was enhanced by deletion of *pka1* (Fig 7). The *Δpka1 Δrad24* strain did not grow on medium containing 0.1 M CaCl_2_, while single deletion mutants of *pka1* or *rad24* grew. We also tried to construct the *Δpka1 rad24E185K* mutant but were unable. Low glucose induced a phenotype similar to that of *pka1* deletion because Pka1 is in an inactive state upon formation of the Pka1-Cgs1 complex under low glucose conditions [10]. When they were grown in 0.1% glucose, *rad24E185K* cells were more sensitive to growth on medium containing 0.1 M CaCl_2_ than on medium containing 3% glucose.

**Fig 8:**
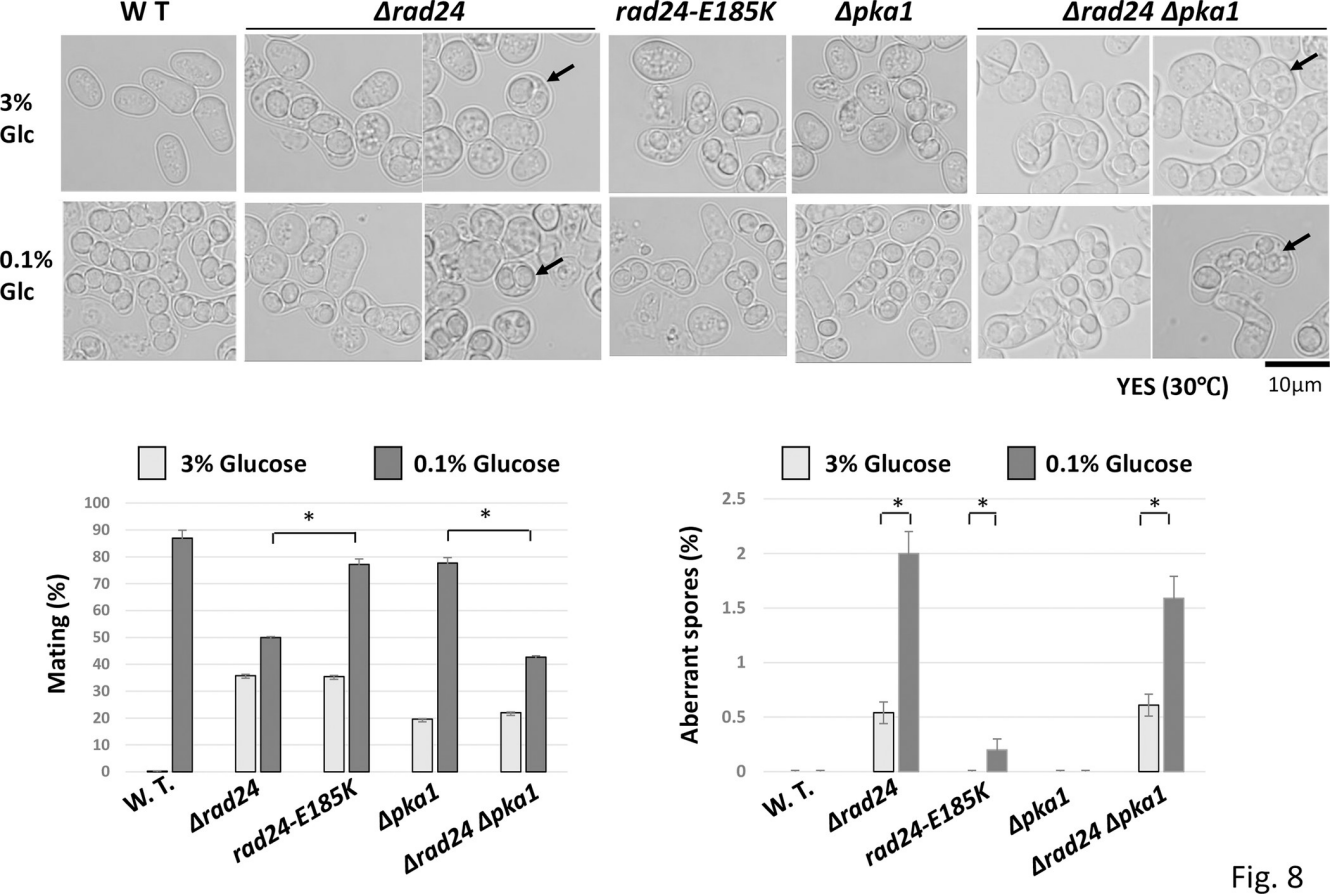
Phenotype of the *rad24 pka1* double deletion mutant. SP870 (*h*^*9li*^WT), TMS1 (*h*^*90*^ Δ*rad24*), TOP3 (*h*^*90*^ *rad24-E185K*), JZ633 (*h*^*90*^ Δ*pka1*), and TOP5 (*h*^*90*^ Δ*rad24* Δ*pka1*) cells were grown in YES medium containing 3% or 0.1% glucose and their normal mating rate and aberrant spores were measured by counting 500 cells. Arrows indicate haploid-derived aberrant 2–4 spores and diploid-derived aberrant 8 spores. Quantification of mating ratio was performed in triplicate and *p* <0.05 (*) was considered statistically significant. The error bars means Standard Deviation (SD) in triplicate samples.

We then measured the mating rate of the *Δpka1 Δrad24* strain. The mating ratio of *Δpka1 Δrad24* (22.0 ± 2.5%) was lower than that of the single deletion mutant of *rad24* (35.8 ± 3.5%) under glucose-rich conditions, but double mutation induced haploid-derived aberrant 2–4 spores and diploid-derived aberrant 8 spores (Fig 8). While the normal mating ratio was decreased in *pka1 rad24* double mutants, it induced uncontrolled meiosis. Induction of this aberrant meiosis was enhanced by low glucose in *Δrad24* (1.9 ± 1.5%) and *Δpka1 Δrad24* (1.6 ± 0.9%). We also found that *rad24-E185K* cells induced haploid meiosis at a lower ratio (0.3 ± 0.2%) than *pka1 rad24* double mutants when cells were grown under low glucose, which created an inactivated state of Pka1.

### Deletion of *rad24* affects Pka1 localization

**Fig 9:**
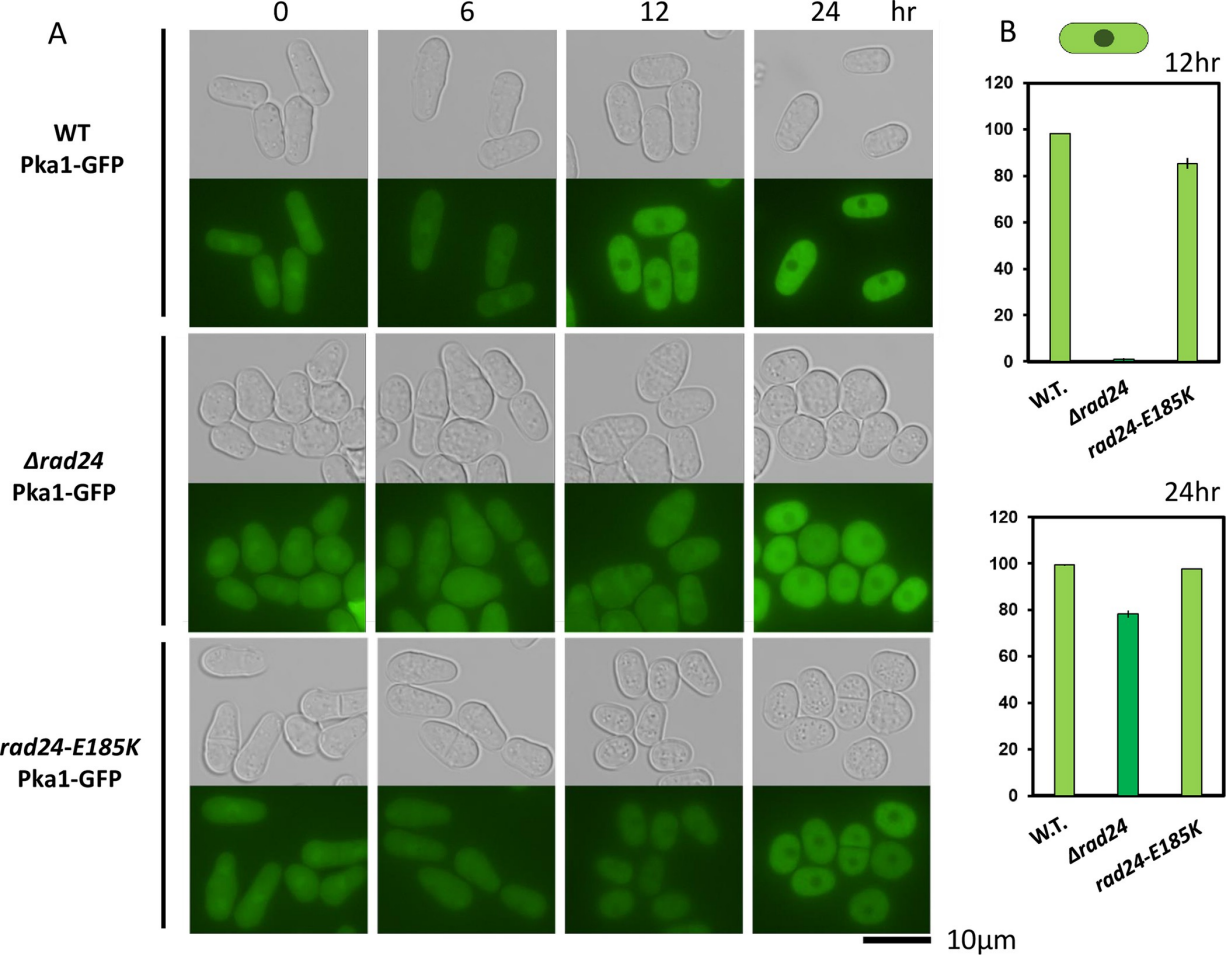
Localization of Pka1-GFP in the *rad24* mutant. YMP27 (Pka1-GFP), TOP10 (Pka1-GFP Δ*rad24*) and TOP11 (Pka1-GFP *rad24-E185K*) cells were grown in EMM5S-N for 24 h. GFP was observed at 0, 6, 12, and 24 h. (B) Quantification of cytoplasmic localization of the GFP signal based on counting 500 cells at 12 h and 24 h in triplicate. Th error bars indicate SD in triplicate samples.

Finally, we explored whether localization of Pka1 was affected by Rad24 by constructing Pka1-GFP *Δrad24* and Pka1-GFP *rad24-E185K* strains. Upon starvation, Pka1 is translocated from the nucleus to the cytoplasm [42]. This localization shift was observed after 12 h of starvation in WT and *rad24-E185K* strains, but not in the *Δrad24* strain (Fig 9). The localization shift of Pka1 in the *Δrad24* strain was observed after 24 h, indicating that Rad24 is required for timely exclusion of Pka1 from the nucleus. The *rad24-E185K* mutation did not affect this timely exclusion of Pka1. Thus, Rad24 is involved in a quick response to Pka1 localization.

## Discussion

Sexual differentiation has been studied extensively in *S*. *pombe*. Among the players regulating this process, Ste11 is a key transcription factor [18] and Mei2 is an RNA-binding protein that ensures the transition of mitosis to meiosis [14]. Ste11 is controlled in many ways: transcription of *ste11* is negatively controlled by transcription factor Rst2 [13], which is under the control of Pka1; its cellular localization is controlled by the Ras-MAP (Byr2-Byr1-Spk1) kinase pathway [19] and is phosphorylated by Pat1 kinase to bind with 14-3-3 [20]; and its translation is controlled by Cpc2-Msa2 [8, 43]. Among the proteins that interact with 14-3-3, Ste11, Mei2, and Byr2 are important regulators of sexual differentiation.

In this study, we explored *sam3* and *sam9* mutants of the fission yeast *S*. *pombe*, isolated as mutants that mate in nutrient-rich medium, and found that both have an *E185K* mutation in *rad24* encoding an 14-3-3 protein. Members of the 14-3-3 family, widely conserved among eukaryotes, play regulatory roles by binding to target proteins [44]. *S*. *pombe* has two 14-3-3 proteins, Rad24 and Rad25, and whereas single deletion of *rad24* or *rad25* does not affect survival, both deletion is lethal [45]. Deletion of *rad24* causes radiation sensitivity; hence, it was named, but this phenotype is not clearly observed in Δ*rad25* cells. The *sam* mutant phenotype, mating and sporulating on rich medium, has been observed in *rad24*, *cyr1*, and *pka1* mutants [3, 10], but not in the Δ*rad25* mutant [27]. The *sam* phenotype of the *rad24-E185K* mutant is similar to that of the *Δrad24* strain, but it differs in terms of sensitivity to UV; while the *Δrad24* strain is sensitive to UV, the *rad24-E185K* mutant is not. This difference between *Δrad24* and *rad24-E185K* mutants is explained by the regulatory mechanism of the Chk1 protein investigated in the present study. Chk1 requires Rad24 for its full activation [34]. Upon deletion of *rad24* Chk1 remains in an inactive state, and normal activation of Chk1 by UV does not occur, which causes UV sensitivity in the *rad24* deletion strain. Because Rad24-E185K still retains the ability to interact with Chk1, as we demonstrated in this work, the regulatory mechanism of Chk1 by Rad24-E185K appears to be normal. Unlike the role of Rad24 in this activation of Chk1, Rad24 negatively regulates Mei2, Ste11, and Byr2; hence, these three proteins remain active in *Δrad24* cells. In the *rad24-E185K* strain, interaction with Mei2, Ste11, and Byr2 was weakened; hence, these three proteins remain in an active state, as in *Δrad24* cells. Therefore, this property of Rad24 accounts for the sexual differentiation phenotype of *rad24-E185K* mutants.

Herein, we showed that haploid meiosis was induced in *rad24* and *pka1* double deletion mutants. Haploid meiosis is induced by hyperactivation of Byr1 [22], the *pat1* mutation [46], the *mei2-SATA* mutation [14], or expression of a truncated version of Sla1 [30]. Given that Rad24 interacts with Byr2, Ste11, and Mei2, and that Pka1 affects the expression of *ste11*, it is reasonable to assume that sexual differentiation is hyperactivated in *rad24* and *pka1* double deletion mutants. Hyperactivation of Byr2, Ste11, and Mei2 occurs in the Δ*rad24* strain, and *ste11* is induced in *pka1*; hence, ectopic meiosis is induced in double deletion mutants, leading to the formation of spores in the haploid state, which decreases normal spore formation. Haploid meiosis is also observed in the *rad24-E185K* under glucose starvation because Pka1 remains in an inactive form under this condition [10].

When *rad24* is deleted, translocation of Pka1 from the nucleus to the cytoplasm upon starvation is delayed. Although we have not studied the mechanism of this regulation, we think a direct regulation of Pka1 by Rad24 is one possibility. Because this regulation is observed in a short term period (0-12h), it may have an meaning other than sexual differentiation.

We previously observed that cAMP levels are lower in *sam3* and *sam9* mutants, but not in other *sam* mutants [26]. We also observed that the N-terminal domain of cyclase-associated protein 1 (Cap1) interacts with Rad24 and Rad25 [47]. Cap1 is necessary for full activation of adenylate cyclase [4] and it interacts with adenylate cyclase via its N-terminal domain [48]; therefore, interaction of this domain of Cap1 with Rad24 or Rad25 inhibits adenylate cyclase activity. Based on this knowledge and the current results, we propose that one of the mechanisms to lower cAMP levels in *sam3* and *sam9* mutants involves the dominant negative effect of Rad24-E185K.

The CaCl_2_-sensitive phenotype of *rad24* cells is explained by the loss of downregulation of Prz1, which controls genes involved in Ca homeostasis [49]. We observed the CaCl_2_-sensitive phenotype in both *rad24Δ* and *rad24-E185K* strains. Therefore, we predicted that Rad24*-*E185K would abolish interaction with Prz1. Double deletion of *rad24* and *pka1* enhanced CaCl_2_ sensitivity. Since Pka1 regulates the expression of Prz1 [50] and since Rad24 interacts with Prz1, this enhancement of CaCl_2_ sensitivity in *rad24* and *pka1* likely reflects the enhancement of two separate regulatory mechanisms of Prz1.

**Fig 10:**
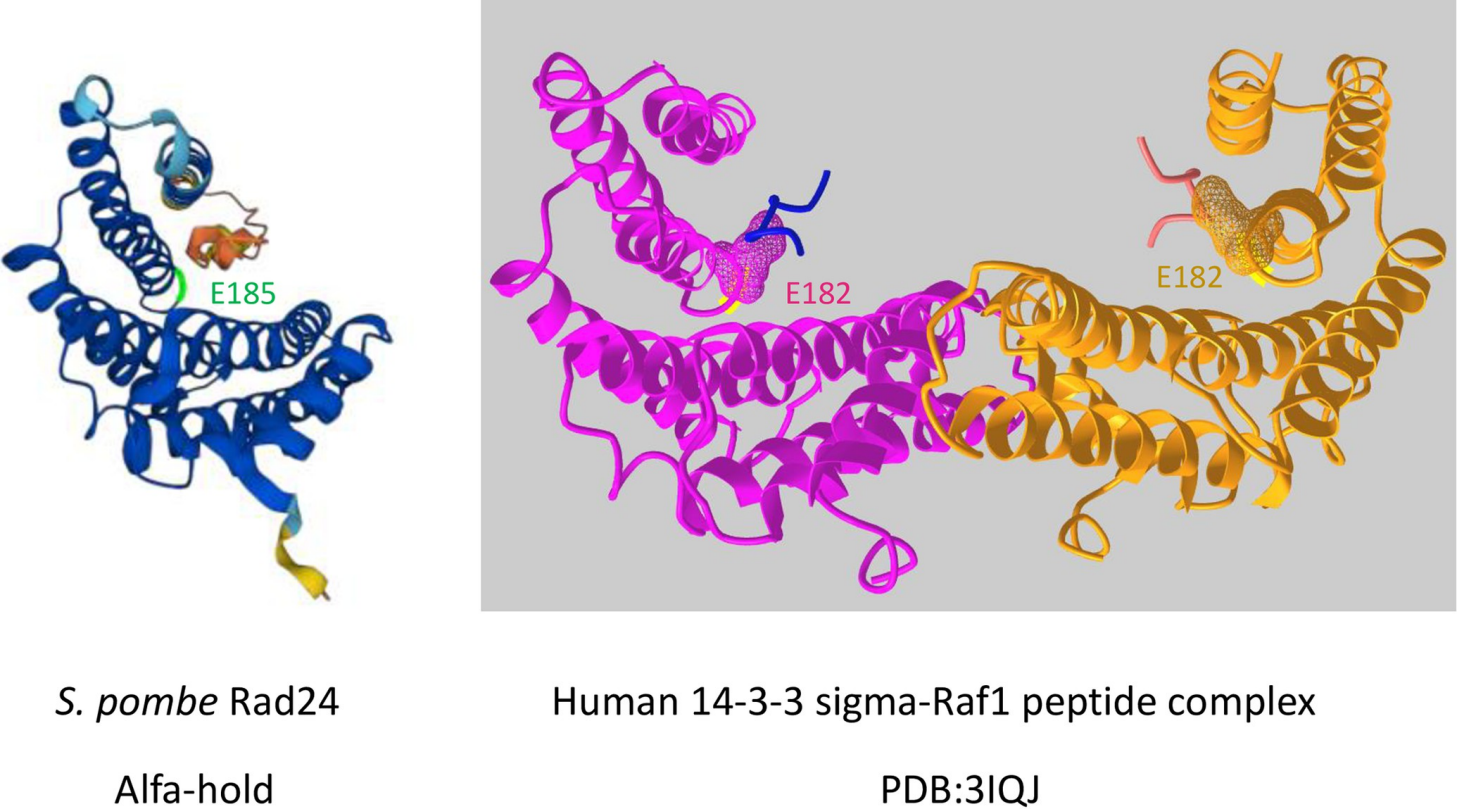
Three-dimensional structure of 14-3-3 proteins. (A) Three-dimensional structure of *S*. *pombe* Rad24 predicted by AlphaFold. (B) Three-dimensional structure of human 14-3-3 sigma co-crystalized with 10 peptides derived from Raf1 (PDB:3IQJ). The position of E185 and the equivalent position of E182 in human 14-3-3 sigma is highlighted.

Amino acid E185 is conserved in 14-3-3 proteins in fungi, animals, and plants. A major of 14-3-3 proteins contain nine alpha-helices, and the structure of Rad24 predicted by AlphaFold [51] resembles the crystal structure of human 14-3-3 sigma (Fig 10). E185 is located at the C-terminal edge of alpha-helix 7, which is located at the site of interaction with other proteins (e.g. mammalian Raf1; an ortholog of Byr2) and proximal to the site of dimer formation of 14-3-3s [52]. Mutational analysis of 14-3-3 zeta to study its interaction with Raf1 has been conducted [53], but mutation of E182 (equivalent to E185 of Rad24) in 14-3-3 zeta has not been analyzed. Our finding that the E185K mutation affected interactions with proteins such as Mei2, Ste11, and Byr2, but not Chk1, indicates that E185 is important for determining the specificities of 14-3-3 protein interactions. Given that E185 is a highly conserved residue located at the site of interaction with target proteins, the *E185K* mutant should provide useful information for studying the dominant negative effects of 14-3-3 in other organisms.

## Supporting information

S1 File (PDF)

S1 Table Primers used in this study. (DOCX)

S1 Data (PDF)

## References

[1] Otsubo Y, Yamamoto M. Signaling pathways for fission yeast sexual differentiation at a glance. J Cell Sci. 2012;125(Pt 12):2789–93. doi: 10.1242/jcs.094771 . 22879382

[2] Welton R, Hoffman C. Glucose monitoring in fission yeast via the gpa2 G alpha, the git5 G beta and the git3 putative glucose receptor. Genetics. 2000;156(2):513–21. WOS:000089766800005. doi: 10.1093/genetics/156.2.513 11014802 PMC1461262

[3] Kawamukai M, Ferguson K, Wigler M, Young D. Genetic and biochemical-analysis of the adenylyl cyclase of *Schizosaccharomyces pombe*. Cell Regulation. 1991;2(2):155–64. WOS:A1991EZ58100006. 1863602 10.1091/mbc.2.2.155 PMC361733

[4] Kawamukai M, Gerst J, Field J, Riggs M, Rodgers L, Wigler M, et al. Genetic and biochemical analysis of the adenylyl cyclase-associated protein, cap, in *Schizosaccharomyces pombe*. Molecular Biology of the Cell. 1992;3(2):167–80. WOS:A1992HD21500004. 1550959 10.1091/mbc.3.2.167 PMC275516

[5] Goldar MM, Nishie T, Ishikura Y, Fukuda T, Takegawa K, Kawamukai M. Functional conservation between fission yeast moc1/sds23 and its two orthologs, budding yeast SDS23 and SDS24, and phenotypic differences in their disruptants. Bioscience Biotechnology and Biochemistry. 2005;69(7):1422–6. doi: 10.1271/bbb.69.1422 WOS:000231055700031. 16041152

[6] Goldar MM, Jeong HT, Tanaka K, Matsuda H, Kawamukai M. Moc3, a novel Zn finger type protein involved in sexual development, ascus formation, and stress response of Schizosaccharomyces pombe. Current Genetics. 2005;48(6):345–55. doi: 10.1007/s00294-005-0028-z WOS:000233497300001. 16273369

[7] Kawamukai M. Isolation of a novel gene, moc2, encoding a putative RNA helicase as a suppressor of sterile strains in Schizosaccharomyces pombe. Biochimica Et Biophysica Acta-Gene Structure and Expression. 1999;1446(1–2):93–101. doi: 10.1016/s0167-4781(99)00071-8 WOS:000081526800008. 10395922

[8] Paul S, Oowatari Y, Kawamukai M. A large complex mediated by Moc1, Moc2 and Cpc2 regulates sexual differentiation in fission yeast. Febs Journal. 2009;276(18):5076–93. doi: 10.1111/j.1742-4658.2009.07204.x WOS:000269366500007. 19682301

[9] Yakura M, Ishikura Y, Adachi Y, Kawamukai M. Involvement of Moc1 in sexual development and survival of Schizosaccharomyces pombe. Bioscience Biotechnology and Biochemistry. 2006;70(7):1740–9. doi: 10.1271/bbb.60088 WOS:000239546200026. 16819157

[10] Gupta DR, Paul SK, Oowatari Y, Matsuo Y, Kawamukai M. Complex Formation, Phosphorylation, and Localization of Protein Kinase A of Schizosaccharomyces pombe upon Glucose Starvation. Bioscience Biotechnology and Biochemistry. 2011;75(8):1456–65. doi: 10.1271/bbb.110125 WOS:000294531200008. 21869531

[11] Gupta D, Paul S, Oowatari Y, Matsuo Y, Kawamukai M. Multistep regulation of protein kinase A in its localization, phosphorylation and binding with a regulatory subunit in fission yeast. Current Genetics. 2011;57(5):353–65. doi: 10.1007/s00294-011-0354-2 WOS:000295085800006. 21879336

[12] Kunitomo H, Higuchi T, Iino Y, Yamamoto M. A zinc-finger protein, Rst2p, regulates transcription of the fission yeast ste11(+) gene, which encodes a pivotal transcription factor for sexual development. Molecular Biology of the Cell. 2000;11(9):3205–17. doi: 10.1091/mbc.11.9.3205 WOS:000089387800028. 10982411 PMC14986

[13] Higuchi T, Watanabe Y, Yamamoto M. Protein kinase A regulates sexual development and gluconeogenesis through phosphorylation of the Zn finger transcriptional activator Rst2p in fission yeast. Molecular and Cellular Biology. 2002;22(1):1–11. doi: 10.1128/MCB.22.1.1-11.2002 WOS:000172686000001. 11739717 PMC134213

[14] Watanabe Y, Yamamoto M. S. pombe mei2+ encodes an RNA-binding protein essential for premeiotic DNA synthesis and meiosis I, which cooperates with a novel RNA species meiRNA. Cell. 1994;78(3):487–98. doi: 10.1016/0092-8674(94)90426-x . 7520368

[15] Aono T, Yanai H, Miki F, Davey J, Shimoda C. Mating pheromone-induced expression of the mat1-Pm gene of Schizosaccharomyces pombe: identification of signalling components and characterization of upstream controlling elements. Yeast. 1994;10(6):757–70. doi: 10.1002/yea.320100607 . 7975894

[16] Watanabe Y, Lino Y, Furuhata K, Shimoda C, Yamamoto M. The S.pombe mei2 gene encoding a crucial molecule for commitment to meiosis is under the regulation of cAMP. EMBO J. 1988;7(3):761–7. doi: 10.1002/j.1460-2075.1988.tb02873.x ; PubMed Central PMCID: PMC454389. 2840284 PMC454389

[17] Otsubo Y, Yamashita A, Ohno H, Yamamoto M. S-pombe TORC1 activates the ubiquitin-proteasomal degradation of the meiotic regulator Mei2 in cooperation with Pat1 kinase. Journal of Cell Science. 2014;127(12):2639–46. doi: 10.1242/jcs.135517 WOS:000338443500005. 24741065

[18] Sugimoto A, Iino Y, Maeda T, Watanabe Y, Yamamoto M. Schizosaccharomyces pombe ste11+ encodes a transcription factor with an HMG motif that is a critical regulator of sexual development. Genes Dev. 1991;5(11):1990–9. doi: 10.1101/gad.5.11.1990 . 1657709

[19] Kjaerulff S, Lautrup-Larsen I, Truelsen S, Pedersen M, Nielsen O. Constitutive activation of the fission yeast pheromone-responsive pathway induces ectopic meiosis and reveals Ste11 as a mitogen-activated protein kinase target. Molecular and Cellular Biology. 2005;25(5):2045–59. doi: 10.1128/MCB.25.5.2045-2059.2005 WOS:000227085700039. 15713656 PMC549357

[20] Kitamura K, Katayama S, Dhut S, Sato M, Watanabe Y, Yamamoto M, et al. Phosphorylation of Mei2 and Ste11 by Pat1 kinase inhibits sexual differentiation via ubiquitin proteolysis and 14-3-3 protein in fission yeast. Developmental Cell. 2001;1(3):389–99. doi: 10.1016/s1534-5807(01)00037-5 WOS:000175301400011. 11702950

[21] Toda T, Shimanuki M, Yanagida M. Fission yeast genes that confer resistance to staurosporine encode an AP-1-like transcription factor and a protein kinase related to the mammalian ERK1/MAP2 and budding yeast FUS3 and KSS1 kinases. Genes Dev. 1991;5(1):60–73. doi: 10.1101/gad.5.1.60 . 1899230

[22] Yamamoto TG, Chikashige Y, Ozoe F, Kawamukai M, Hiraoka Y. Activation of the pheromone-responsive MAP kinase drives haploid cells to undergo ectopic meiosis with normal telomere clustering and sister chromatid segregation in fission yeast. Journal of Cell Science. 2004;117(17):3875–86. doi: 10.1242/jcs.01248 WOS:000223733100017. 15265989

[23] Wang Y, Xu HP, Riggs M, Rodgers L, Wigler M. byr2, a Schizosaccharomyces pombe gene encoding a protein kinase capable of partial suppression of the ras1 mutant phenotype. Mol Cell Biol. 1991;11(7):3554–63. doi: 10.1128/mcb.11.7.3554-3563.1991 ; PubMed Central PMCID: PMC361098. 2046669 PMC361098

[24] Bauman P, Cheng Q, Albright C. The Byr2 kinase translocates to the plasma membrane in a Ras1-dependent manner. Biochemical and Biophysical Research Communications. 1998;244(2):468–74. doi: 10.1006/bbrc.1998.8292 WOS:000072881200027. 9514947

[25] Ozoe F, Kurokawa R, Kobayashi Y, Jeong HT, Tanaka K, Sen K, et al. The 14-3-3 proteins Rad24 and Rad25 negatively regulate Byr2 by affecting its localization in Schizosaccharomyces pombe. Molecular and Cellular Biology. 2002;22(20):7105–19. doi: 10.1128/MCB.22.20.7105-7119.2002 WOS:000178266400015. 12242289 PMC139824

[26] Katayama S, Ozoe F, Kurokawa R, Tanaka K, Nakagawa T, Matsuda H, et al. Genetic analysis of the sam mutations, which induce sexual development with no requirement for nutritional starvation in fission yeast. Bioscience Biotechnology and Biochemistry. 1996;60(6):994–9. doi: 10.1271/bbb.60.994 WOS:A1996UU50500013. 8695917

[27] Oowatari Y, Toma K, Ozoe F, Kawamukai M. Identification of sam4 as a rad24 Allele in Schizosaccharomyces pombe. Bioscience Biotechnology and Biochemistry. 2009;73(7):1591–8. doi: 10.1271/bbb.90103 WOS:000268691600022. 19584544

[28] Jeong HT, Ozoe F, Tanaka K, Nakagawa T, Matsuda H, Kawamukai M. A novel gene, msa1, inhibits sexual differentiation in Schizoscharomyces pombe. Genetics. 2004;167(1):77–91. doi: 10.1534/genetics.167.1.77 WOS:000221851100007. 15166138 PMC1470851

[29] Oowatari Y, Jeong H, Tanae K, Nakagawa T, Kawamukai M. Regulation and role of an RNA-binding protein Msa2 in controlling the sexual differentiation of fission yeast. Current Genetics. 2011;57(3):191–200. doi: 10.1007/s00294-011-0335-5 WOS:000290770300004. 21409593

[30] Tanabe K, Ito N, Wakuri T, Ozoe F, Umeda M, Katayama S, et al. Sla1, a Schizosaccharomyces pombe homolog of the human La protein, induces ectopic meiosis when its C terminus is truncated. Eukaryotic Cell. 2003;2(6):1274–87. doi: 10.1128/EC.2.6.1274-1287.2003 WOS:000187363500014. 14665462 PMC326650

[31] Tanabe K, Tanaka K, Matsuda H, Kawamukai M. Truncated Sla1 induces haploid meiosis through the Pat1-Mei2 system in fission yeast. Bioscience Biotechnology and Biochemistry. 2004;68(1):266–70. doi: 10.1271/bbb.68.266 WOS:000188767200043. 14745200

[32] Yakura M, Ozoe F, Ishida H, Nakagawa T, Tanaka K, Matsuda H, et al. zds1, a novel gene encoding an ortholog of Zds1 and Zds2, controls sexual differentiation, cell wall integrity and cell morphology in fission yeast. Genetics. 2006;172(2):811–25. doi: 10.1534/genetics.105.050906 WOS:000236178800012. 16322512 PMC1456246

[33] Sato M, Watanabe Y, Akiyoshi Y, Yamamoto M. 14-3-3 protein interferes with the binding of RNA to the phosphorylated form of fission yeast meiotic regulator Mei2p. Curr Biol. 2002;12(2):141–5. doi: 10.1016/s0960-9822(01)00654-6 . 11818066

[34] Dunaway S, Liu H, Walworth N. Interaction of 14-3-3 protein with Chk1 affects localization and checkpoint function. Journal of Cell Science. 2005;118(1):39–50. doi: 10.1242/jcs.01570 WOS:000226931500005. 15585577

[35] Moreno S, Klar A, Nurse P. Molecular genetic analysis of fission yeast Schizosaccharomyces pombe. Methods Enzymol. 1991;194:795–823. doi: 10.1016/0076-6879(91)94059-l . 2005825

[36] Tanae K, Horiuchi T, Matsuo Y, Katayama S, Kawamukai M. Histone Chaperone Asf1 Plays an Essential Role in Maintaining Genomic Stability in Fission Yeast. Plos One. 2012;7(1). doi: 10.1371/journal.pone.0030472 WOS:000301703800016. 22291963 PMC3266922

[37] Bähler J, Wu JQ, Longtine MS, Shah NG, McKenzie A, Steever AB, et al. Heterologous modules for efficient and versatile PCR-based gene targeting in Schizosaccharomyces pombe. Yeast. 1998;14(10):943–51. doi: 10.1002/(SICI)1097-0061(199807)14:10&lt;943::AID-YEA292&gt;3.0.CO;2-Y . 9717240

[38] Noguchi C, Garabedian MV, Malik M, Noguchi E. A vector system for genomic FLAG epitope-tagging in Schizosaccharomyces pombe. Biotechnol J. 2008;3(9–10):1280–5. doi: 10.1002/biot.200800140 . 18729046

[39] Matsuo Y, Kishimoto H, Tanae K, Kitamura K, Katayama S, Kawamukai M. Nuclear Protein Quality Is Regulated by the Ubiquitin-Proteasome System through the Activity of Ubc4 and San1 in Fission Yeast. Journal of Biological Chemistry. 2011;286(15):13775–90. doi: 10.1074/jbc.M110.169953 WOS:000289282200095. 21324894 PMC3075721

[40] Laemmli UK. Cleavage of structural proteins during the assembly of the head of bacteriophage T4. Nature. 1970;227(5259):680–5. doi: 10.1038/227680a0 . 5432063

[41] Chen L, Liu T, Walworth N. Association of Chk1 with 14-3-3 proteins is stimulated by DNA damage. Genes & Development. 1999;13(6):675–85. doi: 10.1101/gad.13.6.675 WOS:000079429500007. 10090724 PMC316547

[42] Matsuo Y, McInnis B, Marcus S. Regulation of the subcellular localization of cyclic AMP-dependent protein kinase in response to physiological stresses and sexual differentiation in the fission yeast Schizosaccharomyces pombe. Eukaryot Cell. 2008;7(9):1450–9. Epub 20080711. doi: 10.1128/EC.00168-08 ; PubMed Central PMCID: PMC2547071. 18621924 PMC2547071

[43] Jeong HT, Oowatari Y, Abe M, Tanaka K, Matsuda H, Kawamukai M. Interaction between a negative regulator (Msa2/Nrd1) and a positive regulator (Cpc2) of sexual differentiation in Schizosaccharomyces pombe. Bioscience Biotechnology and Biochemistry. 2004;68(7):1621–6. doi: 10.1271/bbb.68.1621 WOS:000223031900034. 15277777

[44] Obsilova V, Obsil T. Structural insights into the functional roles of 14-3-3 proteins. Front Mol Biosci. 2022;9:1016071. Epub 20220916. doi: 10.3389/fmolb.2022.1016071 ; PubMed Central PMCID: PMC9523730. 36188227 PMC9523730

[45] Ford JC, al-Khodairy F, Fotou E, Sheldrick KS, Griffiths DJ, Carr AM. 14-3-3 protein homologs required for the DNA damage checkpoint in fission yeast. Science. 1994;265(5171):533–5. doi: 10.1126/science.8036497 . 8036497

[46] Iino Y, Yamamoto M. Negative control for the initiation of meiosis in Schizosaccharomyces pombe. Proc Natl Acad Sci U S A. 1985;82(8):2447–51. doi: 10.1073/pnas.82.8.2447 ; PubMed Central PMCID: PMC397575. 16593556 PMC397575

[47] Zhou G, Yamamoto T, Ozoe F, Yano D, Tanaka K, Matsuda H, et al. Identification of a 14-3-3 protein from Lentinus edodes that interacts with CAP (adenylyl cyclase-associated protein), and conservation of this interaction in fission yeast. Bioscience Biotechnology and Biochemistry. 2000;64(1):149–59. doi: 10.1271/bbb.64.149 WOS:000085194300020. 10705460

[48] Yamamoto T, Kobayashi-Ooka Y, Zhou GL, Kawamukai M. Identification and characterization of Csh3 as an SH3 protein that interacts with fission yeast Cap1. Fems Yeast Research. 2015;15(8). doi: 10.1093/femsyr/fov097 WOS:000367341100014. 26542710

[49] Koike A, Kato T, Sugiura R, Ma Y, Tabata Y, Ohmoto K, et al. Genetic screening for regulators of Prz1, a transcriptional factor acting downstream of calcineurin in fission yeast. J Biol Chem. 2012;287(23):19294–303. Epub 20120411. doi: 10.1074/jbc.M111.310615 ; PubMed Central PMCID: PMC3365961. 22496451 PMC3365961

[50] Matsuo Y, Kawamukai M. cAMP-dependent protein kinase involves calcium tolerance through the regulation of Prz1 in Schizosaccharomyces pombe. Bioscience Biotechnology and Biochemistry. 2017;81(2):231–41. doi: 10.1080/09168451.2016.1246171 WOS:000393294200005. 27756188

[51] Jumper J, Evans R, Pritzel A, Green T, Figurnov M, Ronneberger O, et al. Highly accurate protein structure prediction with AlphaFold. Nature. 2021;596(7873):583–9. Epub 20210715. doi: 10.1038/s41586-021-03819-2 ; PubMed Central PMCID: PMC8371605. 34265844 PMC8371605

[52] Molzan M, Schumacher B, Ottmann C, Baljuls A, Polzien L, Weyand M, et al. Impaired binding of 14-3-3 to C-RAF in Noonan syndrome suggests new approaches in diseases with increased Ras signaling. Mol Cell Biol. 2010;30(19):4698–711. Epub 20100802. doi: 10.1128/MCB.01636-09 ; PubMed Central PMCID: PMC2950525. 20679480 PMC2950525

[53] Wang H, Zhang L, Liddington R, Fu H. Mutations in the hydrophobic surface of an amphipathic groove of 14-3-3zeta disrupt its interaction with Raf-1 kinase. J Biol Chem. 1998;273(26):16297–304. doi: 10.1074/jbc.273.26.16297 . 9632690

