# Supplementary material for: A dominant negative 14-3-3 mutant in Schizosaccharomyces pombe distinguishes the binding proteins involved in sexual differentiation and check point
Source: PLoS One. 2023 Oct 3;18(10):e0291524. doi: 10.1371/journal.pone.0291524 (PMC10547172; doi:10.1371/journal.pone.0291524)

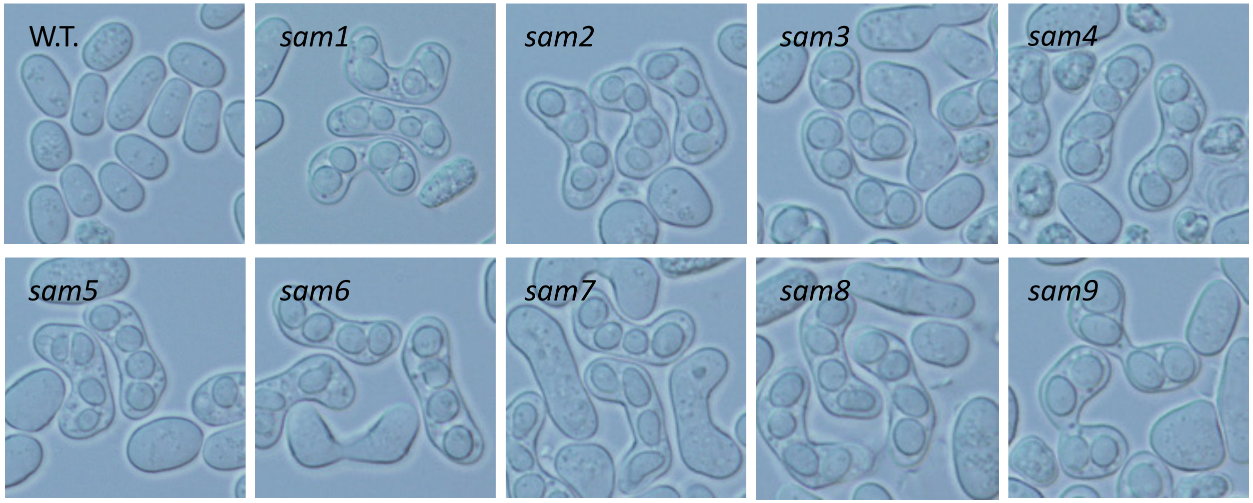

sam : **<u>s</u>**kip the requirement of st**<u>a</u>**rvation for **<u>m</u>**ating

sam1 & sam8 : recessive, *pka1-G359D*
sam2: recessive, *pka1-C358Y*
sam4: recessive, *rad24-Q70**ochre
sam5 : recessive, *pka1* G441E
sam6 : recessive, *pka1*-W461*amber
sam7 : recessive, *pka11*-G441R
sam3 & 9 : dominant, *rad24-E185K*

S1 Fig.

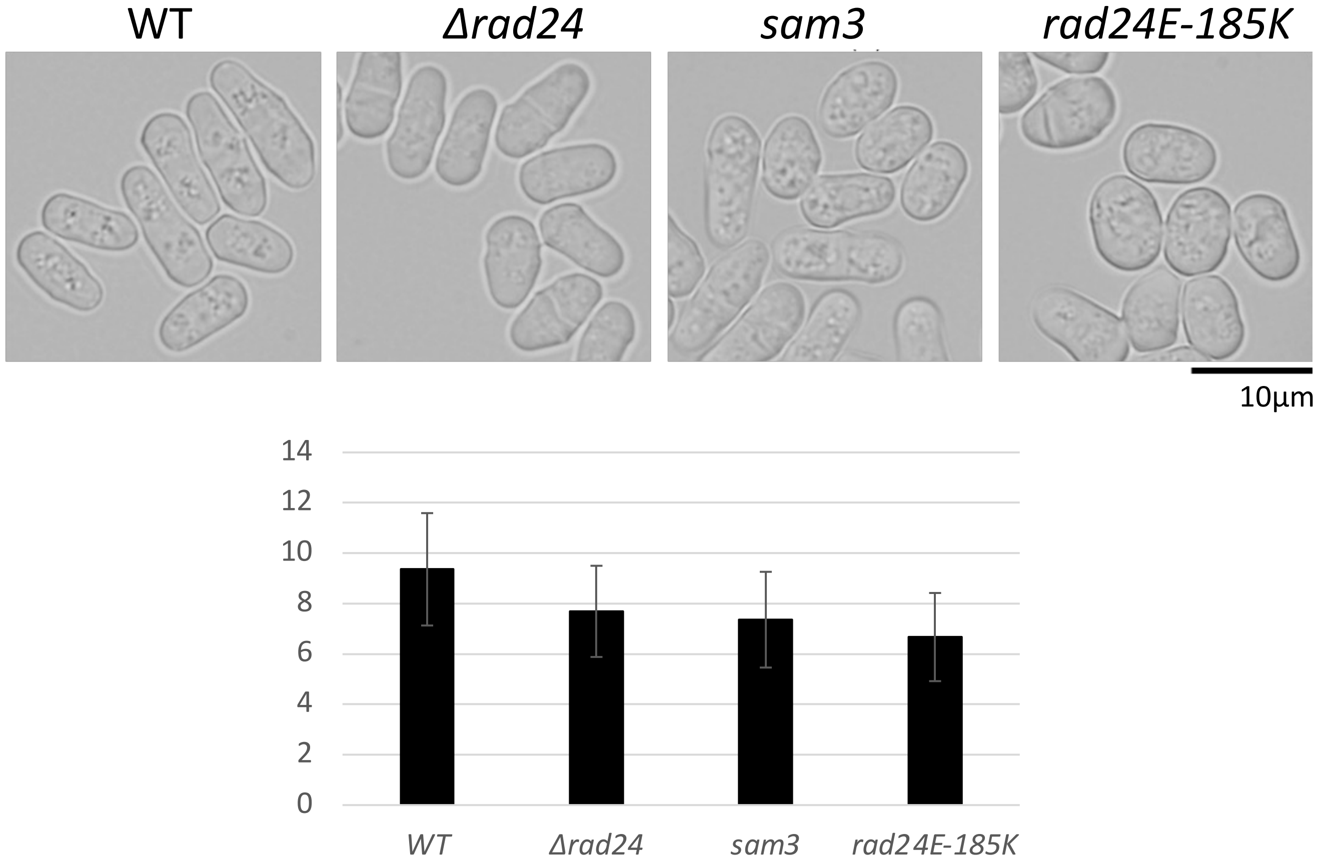
WT
Δrad24
sam3
rad24E-185K
10μm
14
12
10
8
6
4
2
0
WT
Δrad24
sam3
rad24E-185K

S2 Fig.

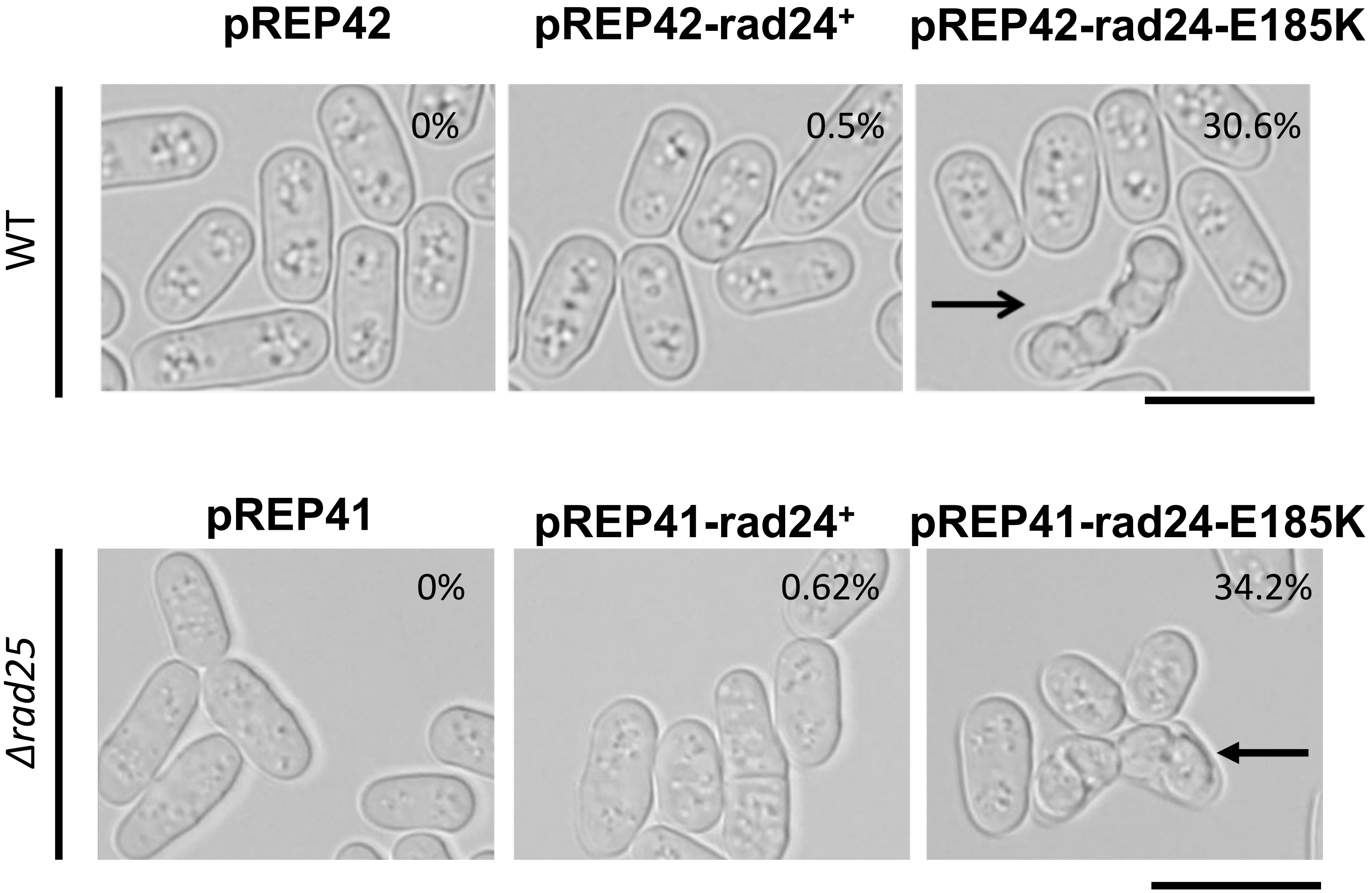
pREP42
pREP42-rad24+
pREP42-rad24-E185K
WT
0%
0.5%
30.6%
pREP41
pREP41-rad24+
pREP41-rad24-E185K
Δrad25
0%
0.62%
34.2%

S3 Fig.

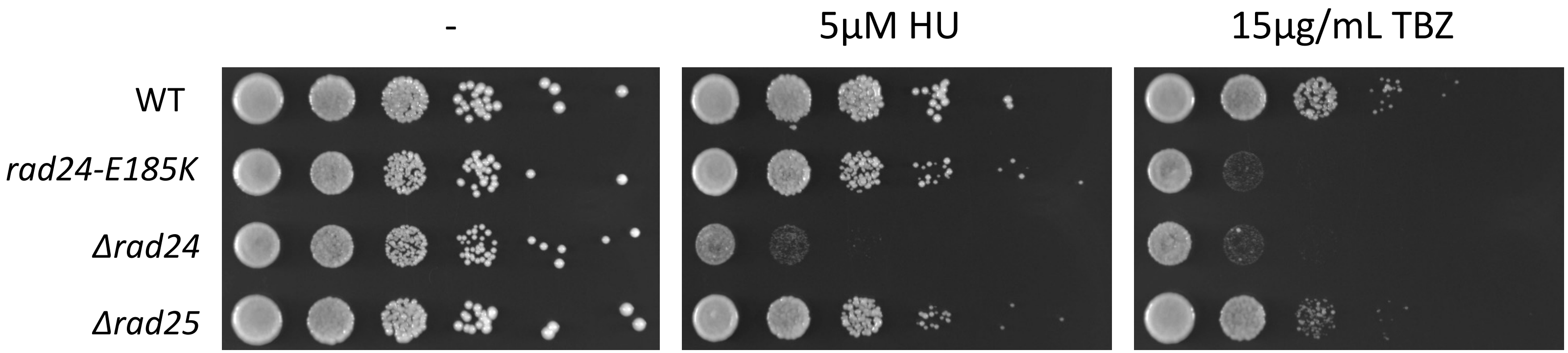

S4 Fig.

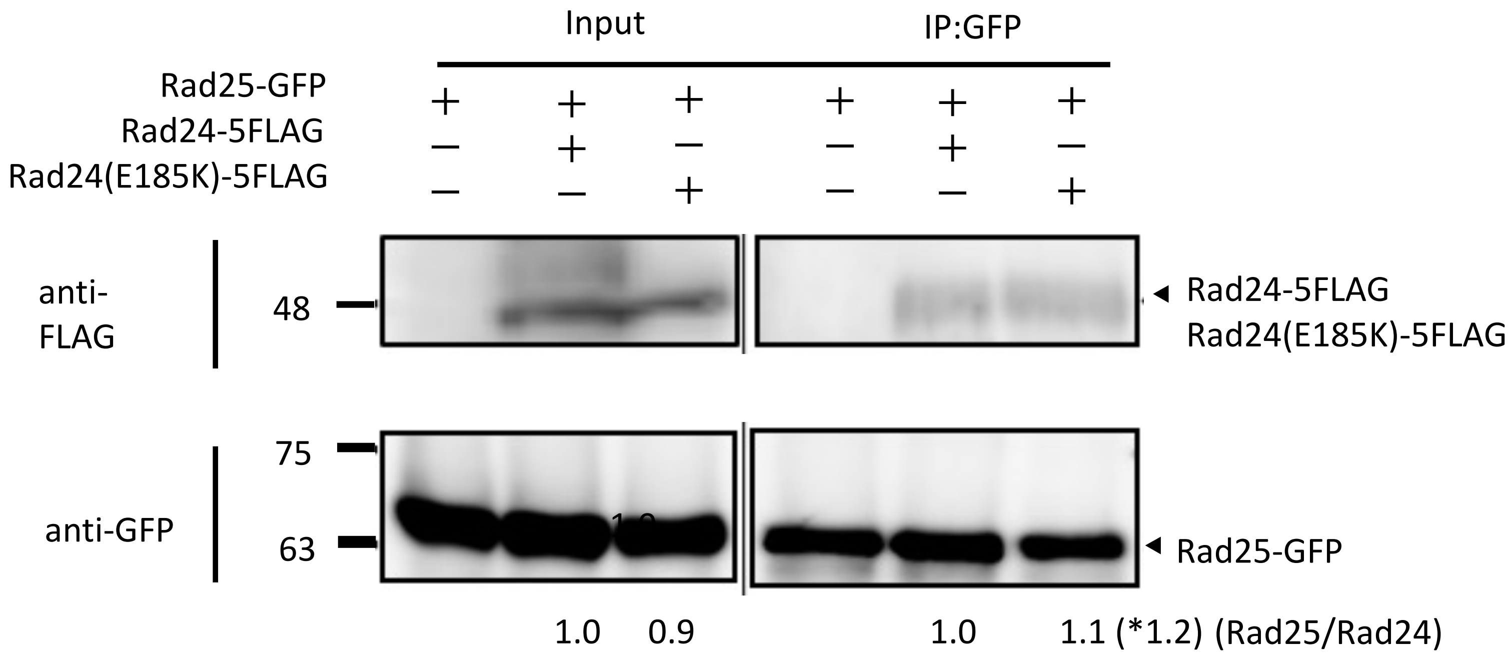
Input
IP:GFP
Rad25-GFP
Rad24-5FLAG
Rad24(E185K)-5FLAG
anti-FLAG
48
Rad24-5FLAG
Rad24(E185K)-5FLAG
anti-GFP
75
63
Rad25-GFP
1.0
0.9
1.0
1.1 (*1.2) (Rad25/Rad24)

S5 Fig.

Raw data of Fig. 4B

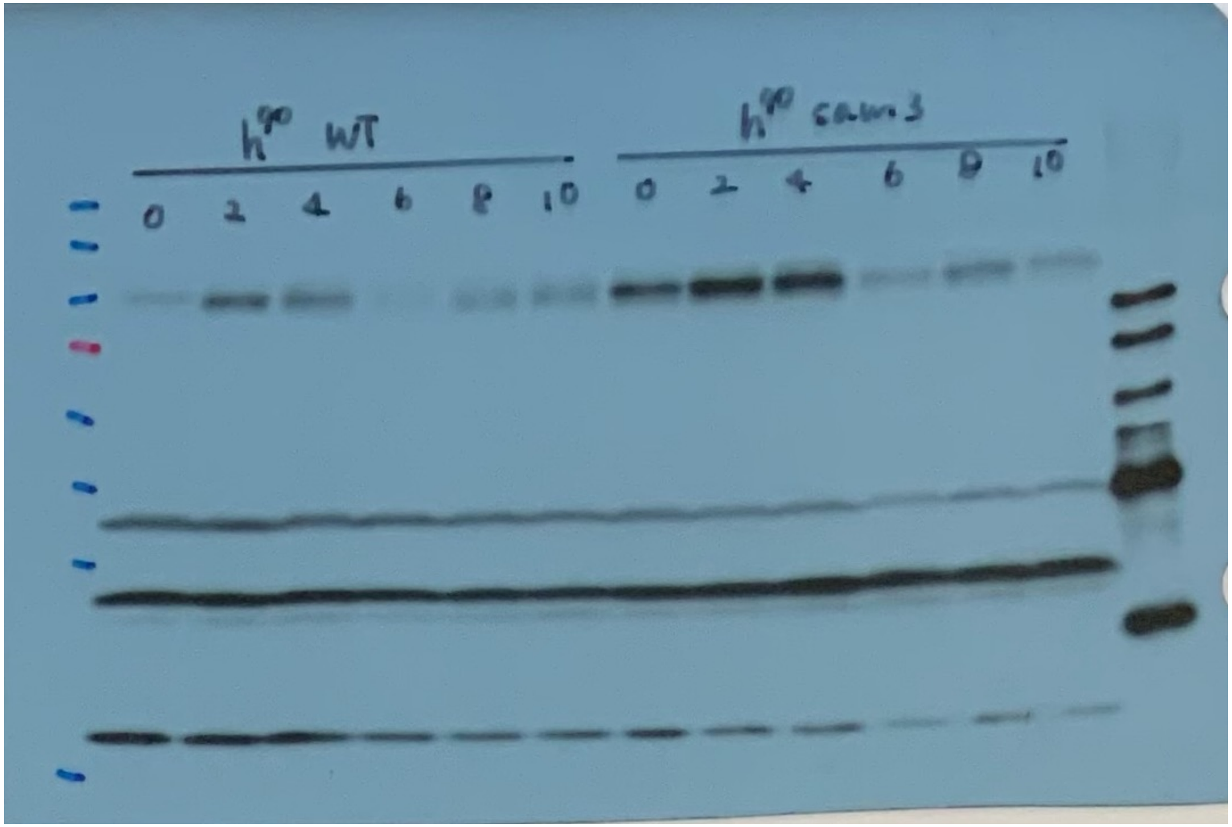

Raw data of Fig. 5

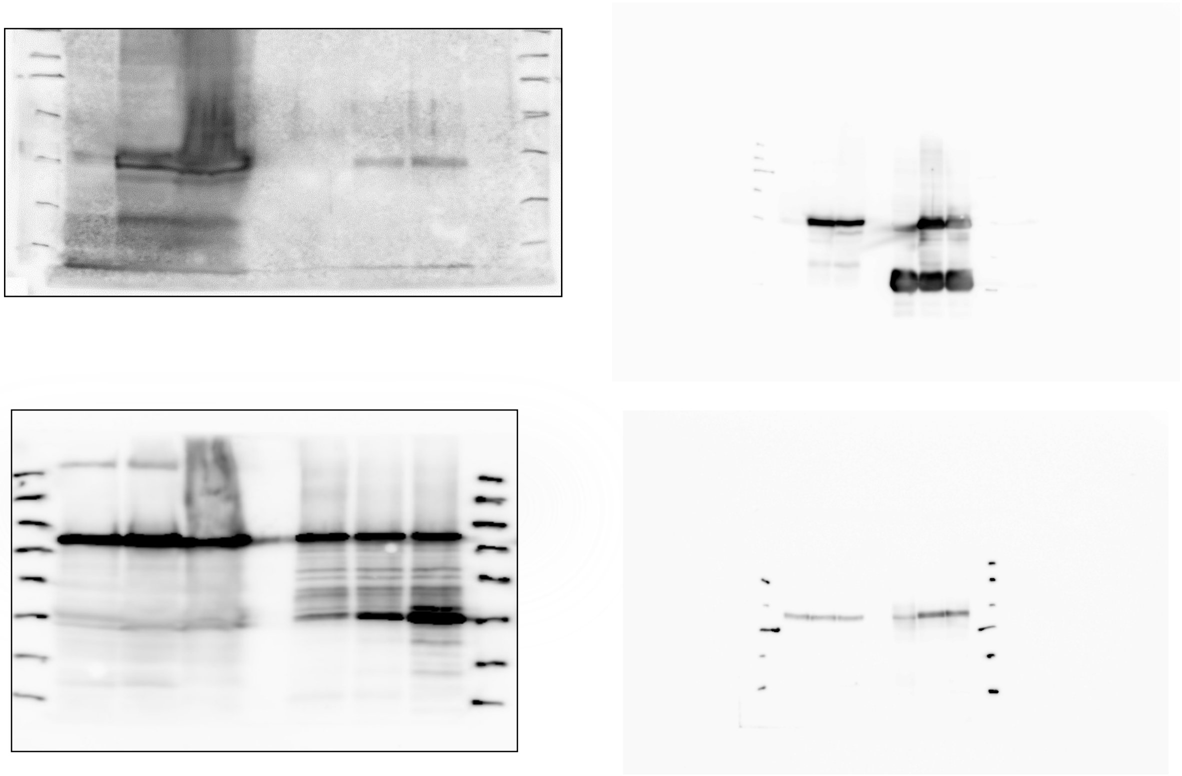

Raw data of Fig. 6

A

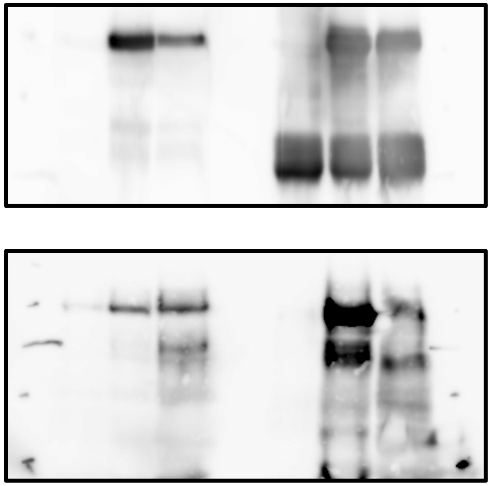

B

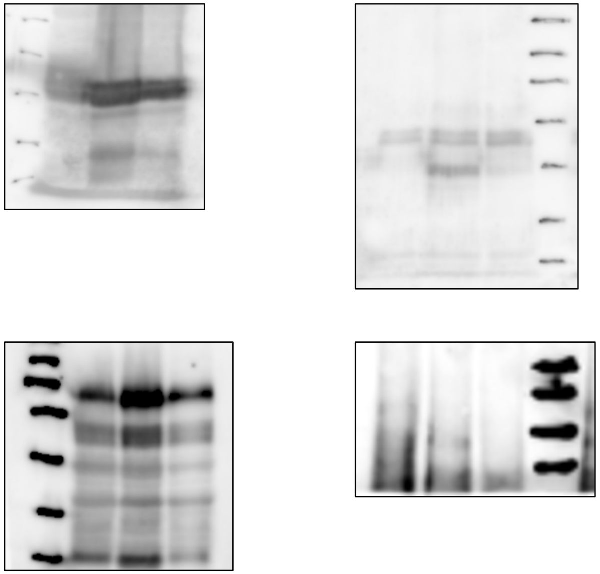

C

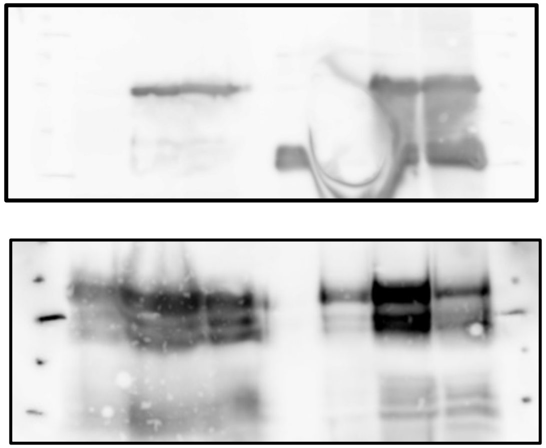

D

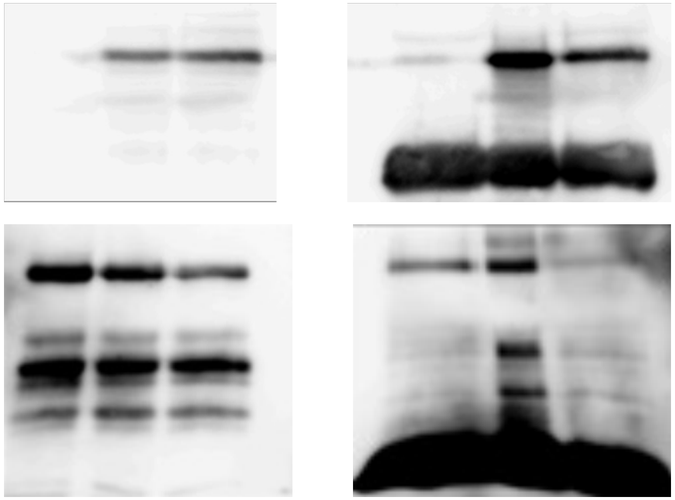

Raw data of Fig. S5

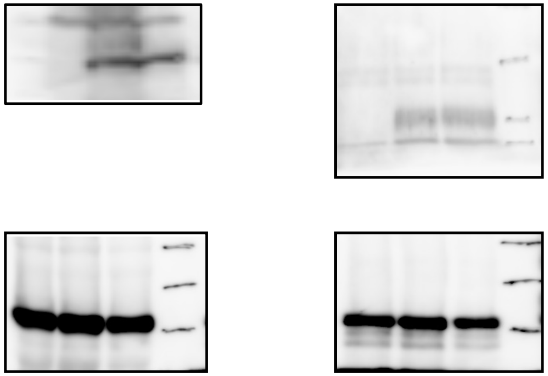

Supplement: S1 File — (PDF) [file pone.0291524.s001.pdf]
