# Supplementary material for: A dominant negative 14-3-3 mutant in Schizosaccharomyces pombe distinguishes the binding proteins involved in sexual differentiation and check point
Source: PLoS One. 2023 Oct 3;18(10):e0291524. doi: 10.1371/journal.pone.0291524 (PMC10547172; doi:10.1371/journal.pone.0291524)
Supplement: S1 Table — (DOCX) [file pone.0291524.s002.docx]

**Table S1 Primers used in this study**

|  |  |
| --- | --- |
| rad24-A | 5’-CCAAGGTTTTCTACTACAAG-3’ |
| rad24-Z | 5’-CAGTCAAAAAGCTCAAATTC-3’ |
| rad24-W | 5’-TAAACGAGCTCGAATTCATCGATCGTTTAGATCTTGTGTACTAC-3’ |
| rad24-B1 | 5’-TCGACCTGCAGCGTACGACAATCATGAGCAACGAGCTATC-3’ |
| rad24-B2 | 5’-TCGACCTGCAGCGTACGATGCGTCCGCCTTGGGCTCAGC-3’ |
| rad24(E185K)-F | 5’-CGTTTTCTACTATAAGATCTTGAAC-3’ |
| rad24(E185K) -R | 5’-GTTCAAGATCTTATAGTAGAAAACG-3’ |
| rad24-check | 5’-CTGTGCGTTTTATATGCAAAC-3’ |
| nb2 | 5’-GTTTAAACGAGCTCGAATTCATCGAT-3’ |
| Ste11-w | 5’-CCCCGAATACCGGTGATC-3’ |
| Ste11-X | 5’-GGGGATCCGTCGACCTGCAGCGTACGAAAATAAATTAGAATTGGG-3’ |
| Ste11-Y | 5’-GTTTAAACGAGCTCGAATTCATCGATCGCGTCACAAAAGCATGC-3’ |
| Ste11-Z | 5’-CGGGCCCAAATTAGATGAC-3’ |
| rad24-PrimerF (*Sal*I) | 5’-GATGTCGACATGTCTACTACTTCTCGTGA-3’ |
| rad24-PrimerR (*Bam*HI) | 5’-CTAGGATCCCTATGCGTCCGCCTTGGGCTCAG-3’ |
| pFA6a-com5 | 5’-TCGTACGCTGCAGGTCGA-3’ |
| pFA6a-com6 | 5’- ATCGATGAATTCGAGCTCGTTTA-3’ |
| rec12- A | 5’-AAccgcttgatctatggctc-3’ |
| rec12- B | 5’-GGGGATCCGTCGACCTGCAGCGTACGACTTTCTGGACGCGTTCAGC-3’ |
| rec12- Y | 5’-GTTTAAACGAGCTCGAATTCATCGATCCTCCAACATGGATGACTTG3’ |
| rec12- Z | 5’-GGTAAACTGACAGGCTGATG-3’ |

*Underlines indicate sequences complementary to *rad24*
