# Supplementary figures and images for: A dominant negative 14-3-3 mutant in Schizosaccharomyces pombe distinguishes the binding proteins involved in sexual differentiation and check point
Source: PLoS One. 2023 Oct 3;18(10):e0291524. doi: 10.1371/journal.pone.0291524 (PMC10547172; doi:10.1371/journal.pone.0291524)

Raw data of Fig. 3B

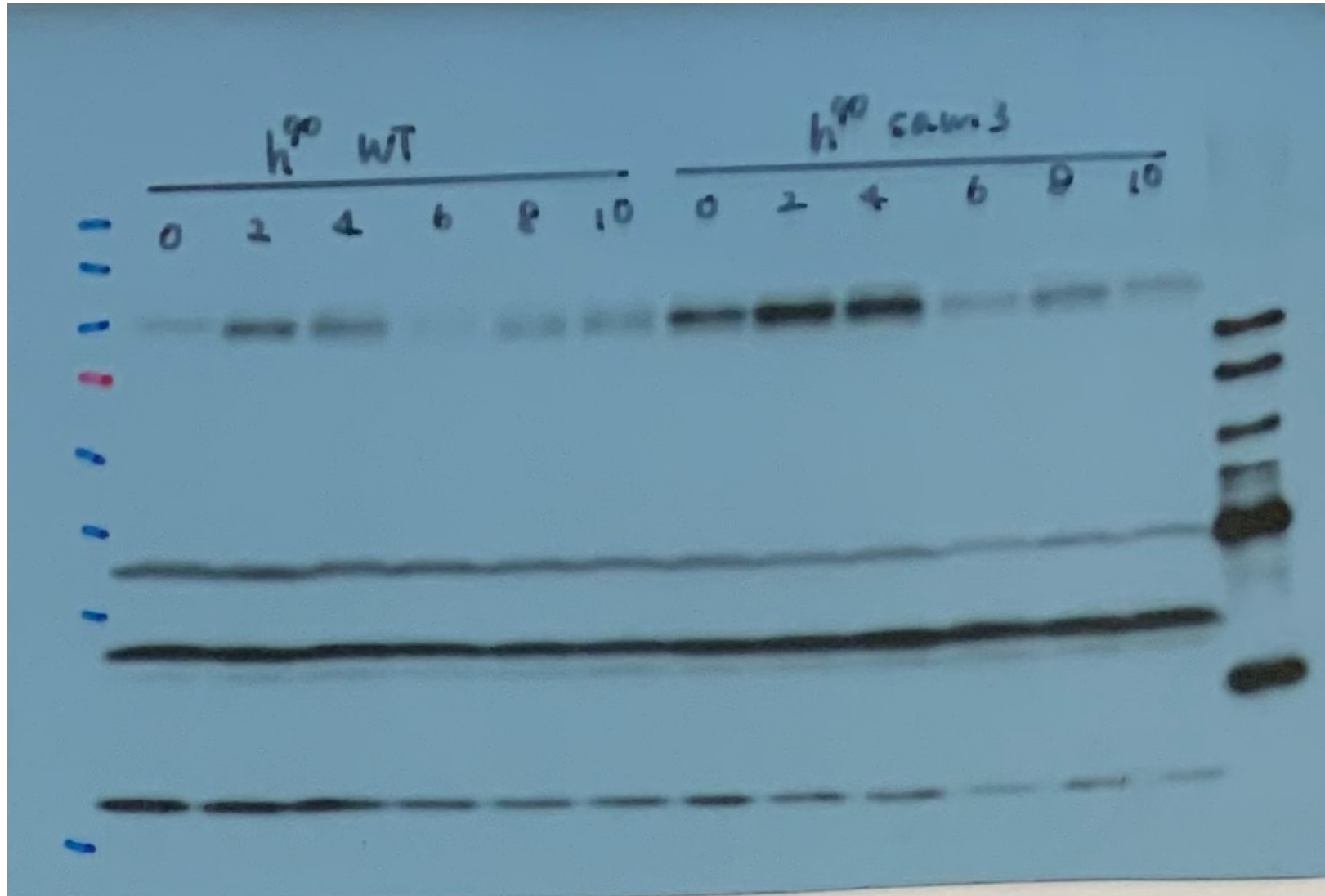

Raw data of Fig. 5

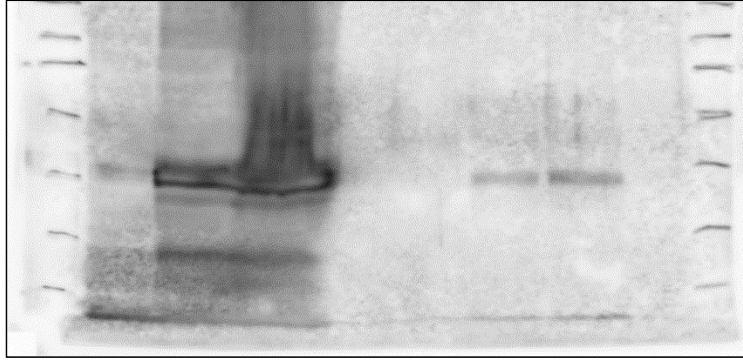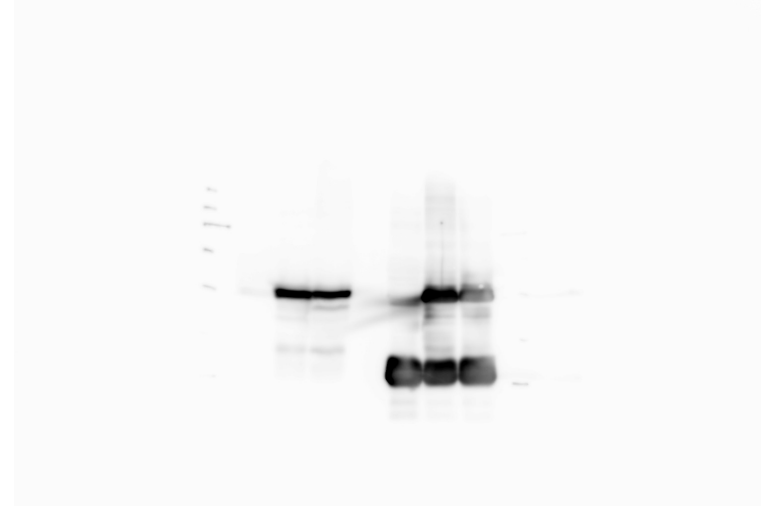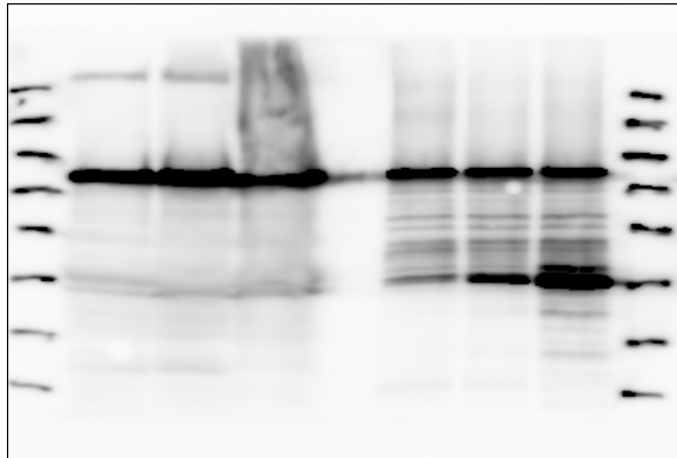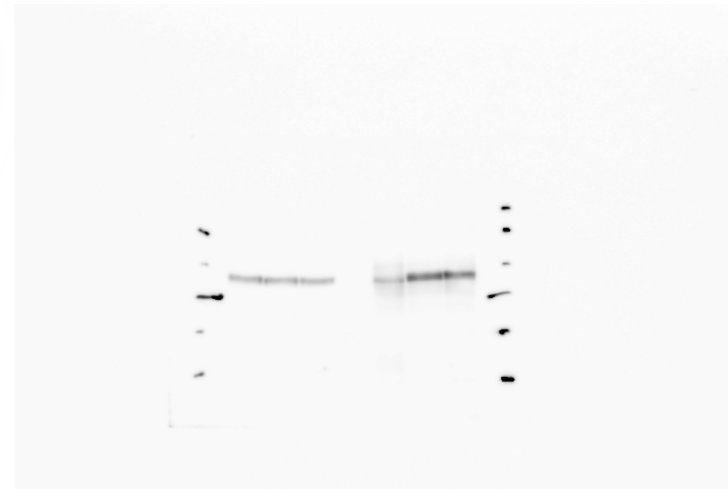

Raw data of Fig. 6

A

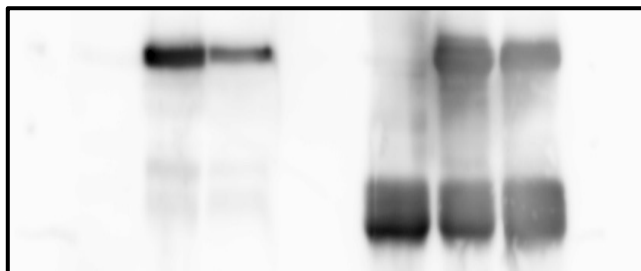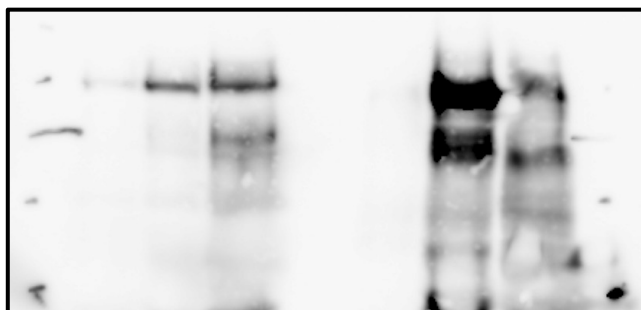

B

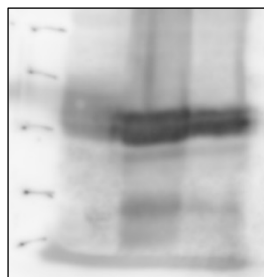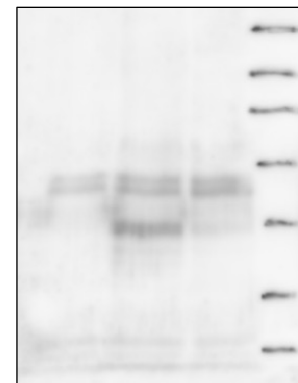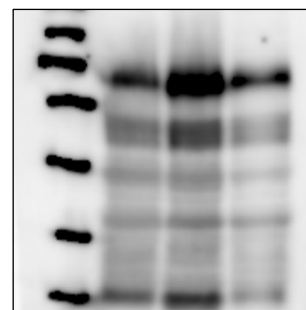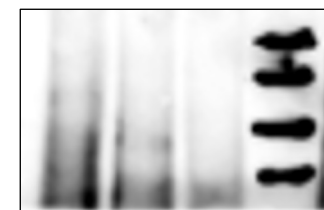

Raw data of Fig. 6

C

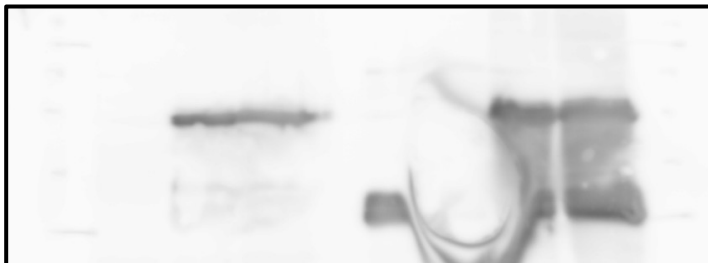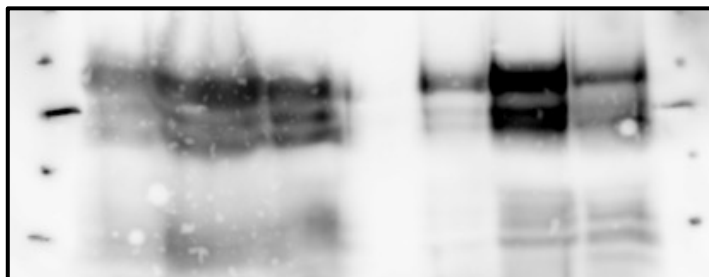

D

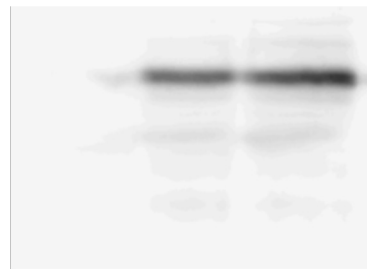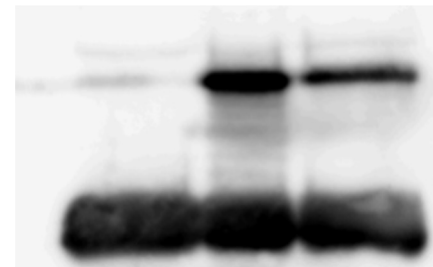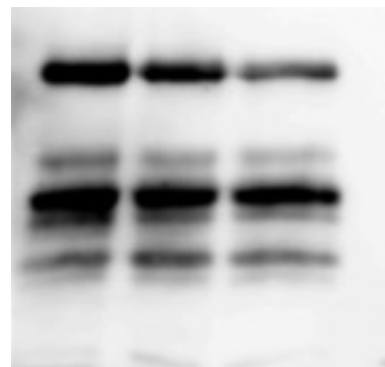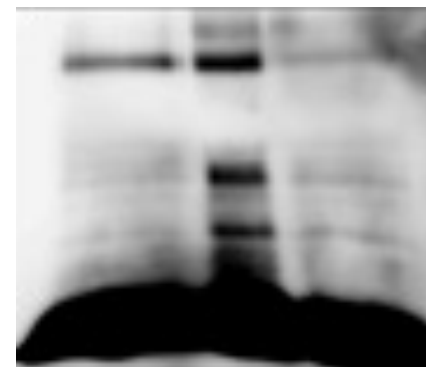

## Raw data of Fig. S5

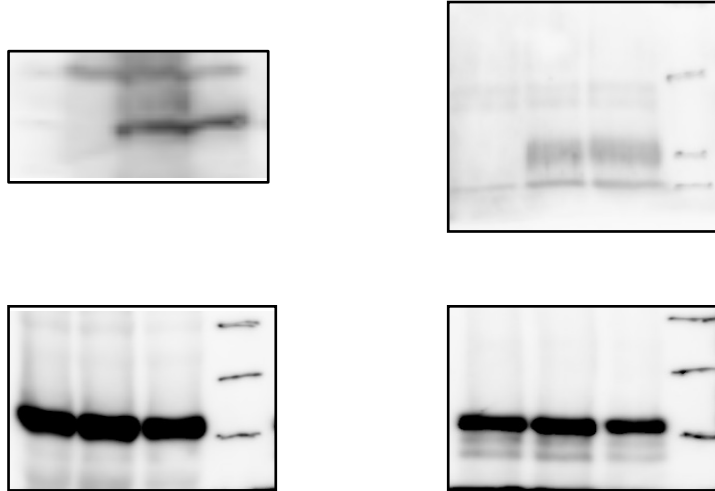

Supplement: S1 Data — (PDF) [file pone.0291524.s003.pdf]
